# One-step Synthesis of Ordered Pd@TiO_2_ Nanofibers Array Film as Outstanding NH_3_ Gas Sensor at Room Temperature

**DOI:** 10.1038/s41598-017-15319-3

**Published:** 2017-11-07

**Authors:** Hongyuan Wu, Haitao Huang, Jiao Zhou, Dahai Hong, Muhammad Ikram, Afrasiab Ur Rehman, Li Li, Keying Shi

**Affiliations:** 1 Key Laboratory of Functional Inorganic Material Chemistry, Ministry of Education. School of Chemistry and Material Science, Heilongjiang University, Harbin, 150080 P.R. China; 2 Key Laboratory of Chemical Engineering Process & Technology for High-efficiency Conversion, School of Chemistry and Material Science, Heilongjiang University, Harbin, 150080 P. R. China; 3 College of Chemistry and Chemical Engineering, Qiqihar University, Qiqihar, 161006 P.R. China; 4 The third affiliated hospital of Qiqihar Medical University, Qiqihar, 161006 P.R. China

## Abstract

The one dimensional (1D) ordered porous Pd@TiO_2_ nanofibers (NFs) array film have been fabricated via a facile one-step synthesis of the electrospinning approach. The Pd@TiO_2_ NFs (PTND3) contained Pd (2.0 wt %) and C, N element (16.2 wt %) display high dispersion of Pd nanoparticles (NPs) on TiO_2_ NFs. Adding Pd meshed with C, N element to TiO_2_ based NFs might contribute to generation of Lewis acid sites and Brønsted acid sites, which have been recently shown to enhance NH_3_ adsorption-desorption ability; Pd NPs could increase the quantity of adsorbed O_2_ on the surface of TiO_2_ based NFs, and accelerated the O_2_ molecule-ion conversion rate, enhanced the ability of electron transmission. The response time of PTND3 sensor towards 100 ppm NH_3_ is only 3 s at room temperature (RT). Meantime, the response and response time of the PTND3 to the NH_3_ is 1 and 14s even at the concentration of 100 ppb. Therefore, the ordered Pd@TiO_2_ NFs array NH_3_ sensor display great potential for practical applications.

## Introduction

NH_3_ sensor is of paramount importance in explosives detection, since it is a decomposition product of many explosive formulations and improvised explosive devices. It is also very useful in monitoring pollutants emitted by inductrial and recycling processes.

Semiconducting metal oxides (SMO) are frequently used as gas-sensing materials. The suitability of a SMO material as a chemiresistor depends on several factors, many of which are correlated with each other. The most obvious factor is the chemical composition. Among the variety of appropriate materials, TiO_2_, as an important non-toxic semiconductor, shows wide potential applications in the sensing field owing to their advantages of earth abundance, chemical and thermal stability^1–5^. Thus, TiO_2_-based gas sensors have also been explored quite extensively. Recent years, 1D ordered porous arrays have been the subject of extensive research due to their distinctive structural features and superior performances^6,7^. However, However, there are still few applications of 1D ordered porous arrays in gas sensing. Chemiresistor arrays with pattern recognition techniques has been demonstrated to be powerful for addressing some of the challenging issues in chemical gas sensor^8^. TiO_2_ nanotubes and nano-fibers are very extensive used for sensing purposes. TiO_2_ nanotubes synthesized by anodization^1,4,6,7,9–15^ and electrospun^16–18^ have higher ordered arrays, higher surface area, and desired location compared with electrospun TiO_2_ nano-fibers, however, ordered template synthesis by anodization is a crucial and tedious technique compared with preparation methods concise and template-free electrospun TiO_2_ nano-fibers and nanotubes. Not only that, the longer electrospun TiO_2_ nano-fibers are in favour of preparing sensing device directly but also a lots of studies which detected gas sensitivity employing some apparatus (such as micro-hotplates^19–21^ and so on) at high temperature have gained certain effect, operating at room temperature becomes more satisfied with commercial development prospect. In previous works, p-type TiO_2_ has been researched^22–24^ and In_2_O_3_/TiO_2_ composite NFs were prepared in our group by electrospinning and exhibited better sensing performance for NO_x_ at RT^25,26^. Nevertheless, the sensors of as prepared In_2_O_3_/TiO_2_ composite nanofiber frequently randomly dispersed on electrodes, which increased resistance noise but the response time was relatively slow. This has been a serious limitation for the potential use of these sensors in applications.

It is well known, that the gas response of the sensor can often be significantly enhanced by chemical modification of the metal oxide’s surface^27–29^. In particular, finely dispersed clusters of noble metals (such as Pt, Pd, Au) may serve as catalysts for the chemical reaction between the analyze gas molecules with semiconductor^30–35^. The electronic and/or chemical interaction between the noble metals with main sensing material is the common reason to increase the selectivity of material and lower the operating temperature^27,36^. while the study on the combined effect of the noble metal with the main sensing material and the auxiliary elements of C, N is comparatively rare.

**Figure 1:**
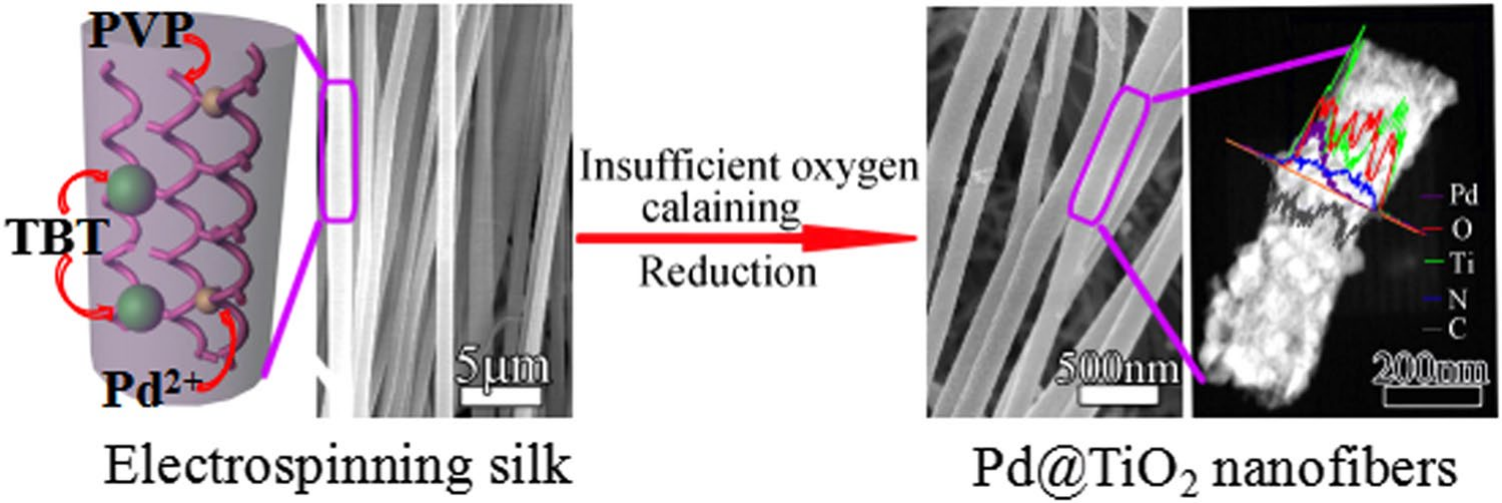
Scheme Design 1D sensitive Pd@TiO_2_ NFs NH_3_ sensor.

In this paper, we suggested a one-step method to fabricate 1D ordered porous Pd@TiO_2_ NFs array film, with remaining small fraction of C, N element under insufficient oxygen calcaining (see Fig. 1). The present studies have provided insights into the interaction of Pd NPs with TiO_2_, and our studies further investigated the increased sensing properties on NH_3_ which was the result of formation of acid sites. And Pd NPs as well as C, N element on the substrate of TiO_2_ might play an important role in the generation of Lewis acid sites and Brønsted acid sites, which can greatly improve the NH_3_ adsorption-desorption ability of gas sensing materials. Therefore, high-powered Pd@TiO_2_ NFs NH_3_ sensor displays great potential for practical applications.

## Results

### Structure and morphology of samples

**Figure 2:**
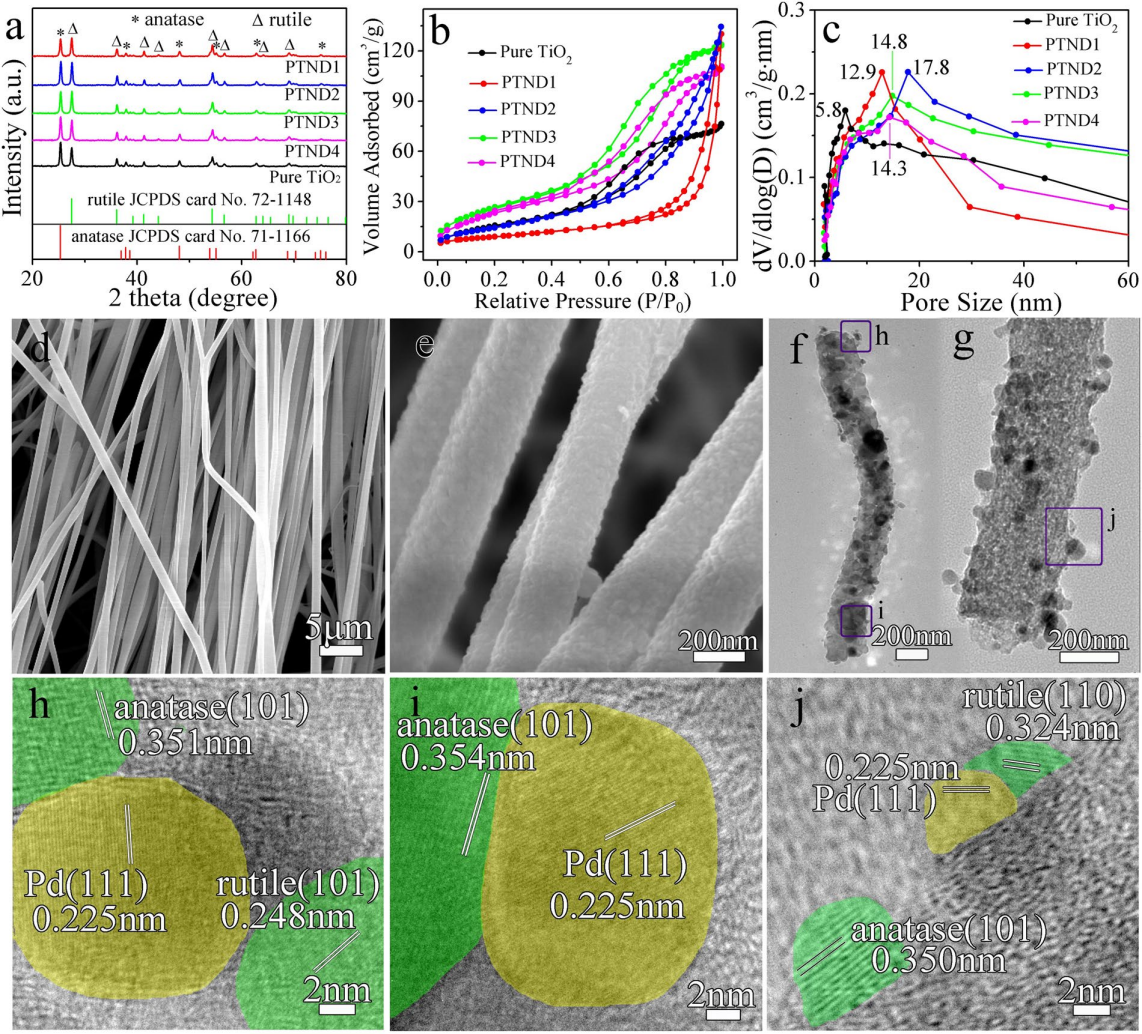
(**a**) XRD patterns; (**b**) N_2_ adsorption-desorption isotherms and (**c**) pore-size distribution curves of pure TiO_2_ and Pd@TiO_2_ NFs; (**d**), (**e**) SEM images of PTND3; (**f**), (**g**) TEM images of PTND3; (**h**), (**i**) and (**j**) HRTEM images of PTND3.

The XRD patterns of pure TiO_2_ and Pd@TiO_2_ NFs are shown in Fig. 2a. It is obvious that the peaks at 2θ values of 25.3, 37.8, 48.0, 55.1, 62.7 and 75.1° can be indexed to (101), (004), (200), (211), (204) and (215) crystal planes of anatase (JCPDS no. 71–1166)^37^, and the peaks at 2θ values of 27.4, 36.1, 41.2, 44.0, 54.3, 56.6, 64.0 and 69.0° can be indexed to (110), (101), (111), (210), (211), (220), (310) and (301) crystal planes of rutile (JCPDS no. 72–1148)^38^, respectively. Meanwhile, XRD patterns of pPTND3 NRs is shown in Fig. S1. Notably, no typical diffraction peaks belonging to Pd (Pd or PdO) are observed in all of the samples, and it is indicated that Pd species are low mass loading and highly dispersed on the TiO_2_ NFs.

Figure 2b,c show the N_2_ adsorption-desorption isotherm and the BET pore-size distribution for samples. The isotherms of samples show a typical type IV with H3-type (PTND1 and PTND2) and H4-type (pure TiO_2_, PTND3 and PTND4) hysteresis loop, which is characteristic of mesoporous material^39^. The BET surface areas and the dominated mesopores are 58.8 m^2^·g^−1^ and 5.8 nm for pure TiO_2_; 82.5 m^2^·g^−1^ and 12.9 nm for PTND1; 86.7 m^2^·g^−1^ and 17.8 nm for PTND2; 100.8 m^2^·g^−1^ and 14.8 nm for PTND3; 89.0 m^2^·g^−1^ and 14.3 nm for PTND4, respectively. It is shows that the BET surface areas and the dominated mesopores results of Pd@TiO_2_ NFs increase significantly than that of pure TiO_2_^40^. This illustrates that it is effective to improve cavity and interface structure with the addition of Pd NPs.

The morphology of calcined Pd@TiO_2_ NFs are investigated by SEM. As shown in Fig. 2d,e and Fig. S2a,b,c, it can be clearly seen that 1D nanofibers of orderly arrangement are obtained on a large scale, which have typical lengths about 5–50 μm, and the diameter about 250 nm.

TEM investigations (Fig. 2f,g,h,i,j) are conducted for further analysis the structure of the PTND3 NFs. PTND3 NFs are consisted of Pd and TiO_2_ polycrystalline nanoparticles as shown in Fig. 2h,i,j. During the calcination process, decomposition of the PVP polymer and Ti-O-Ti network results in the formation of porous and loosened structured of PTND3. The corresponding selected area electron diffraction (SAED) pattern shown in Fig. S3 confirms highly crystalline nature of anatase (101), (004) and rutile (101), (110) planes. HRTEM images in Fig. 2h,i,j confirm that the prepared porous PTND3 NFs have high crystalline nature with the lattice spacing of 0.350 (~0.354) nm corresponding to (101) plane of anatase, and 0.324 and 0.248 nm corresponding to (110) and (101) planes of rutile. Meanwhile, it can be clearly observed the existence of the lattice spacing of 0.225 nm corresponding to (111) planes of Pd NPs^41^, which is illustrated that Pd NPs have been placed into the nanofibers successfully. TEM and HRTEM images of pPTND3 are displayed in Figs S3 and S4, they are also explained that there are anatase, rutile and Pd NPs in the pPTND3. Elemental mapping images of the PTND3 NFs are shown in Fig. S5. Fig. S5 shows a uniform distribution of five elements (Ti, Pd, O, C and N) within the sample.

**Figure 3:**
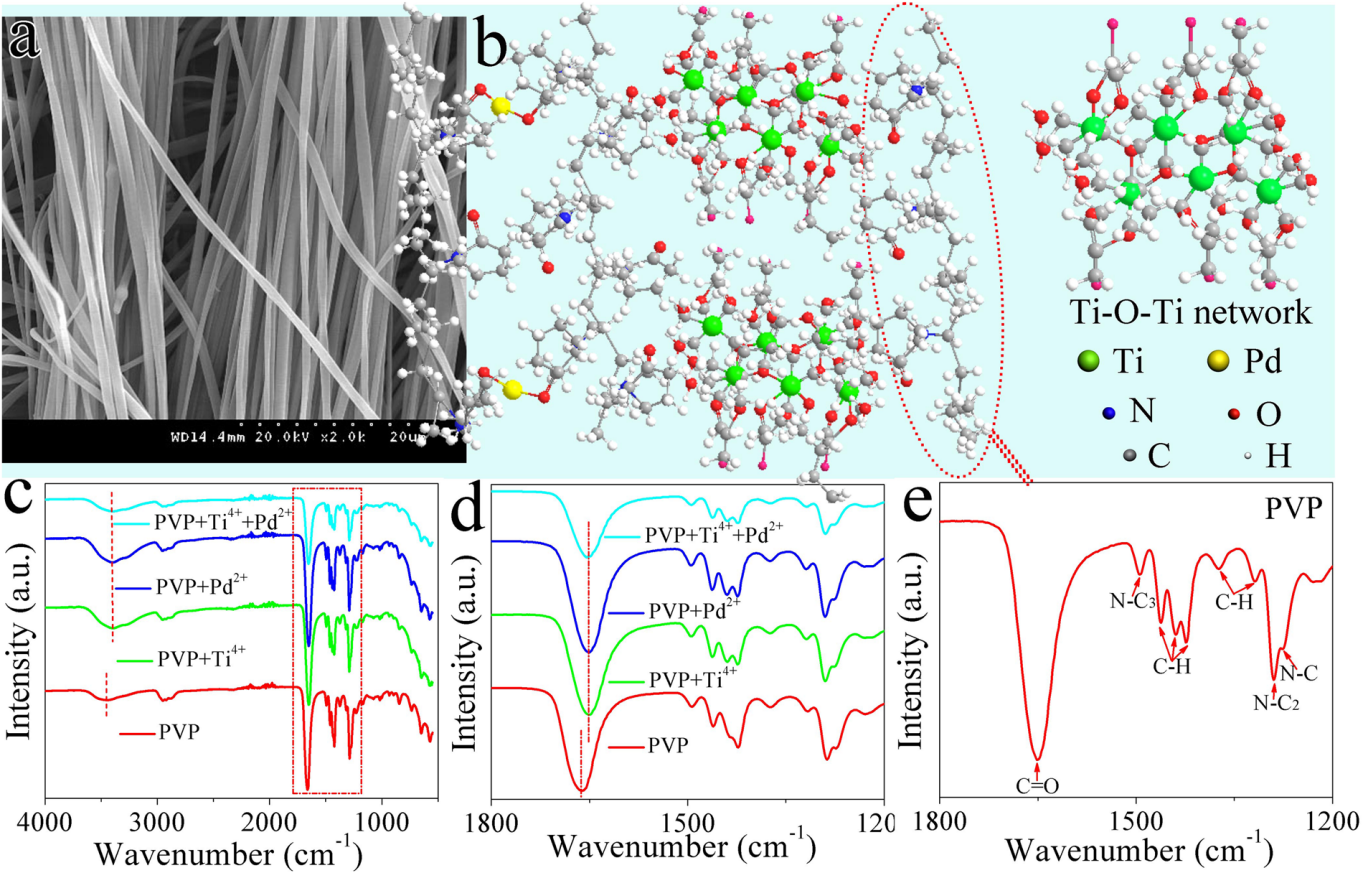
(**a**) SEM image of electrospinning PTND3 NFs array after calcination, (**b**) The proposed crosslinking mechanism of the formation of electrospinning PTND3 NFs (PVP is displayed in red break line circle), (**c**) IR spectra of samples with wavenumber from 400 to 4000 cm^−1^ and (**d**) from 1200 to 1800 cm^−1^, which precursor solution contains of PVP-H_2_PdCl_4_-TBT, PVP-TBT, PVP-H_2_PdCl_4_ and PVP, respectively. (**e**) IR spectra of PVP.

IR spectroscopy (Fig. 3c,d,e) of nanofibers before calcination which precursor solution contains of PVP-H_2_PdCl_4_-TBT, thus, PVP-TBT, PVP-H_2_PdCl_4_ and PVP were measured. Contrast with the IR spectroscopy of pure PVP which contains amide group (N−C stretch of N−C=O (N−C_3_) 1499 cm^−1^), C=O (1659 cm^−1^) and C–N stretch (N−C stretch of N−CH_2_ (N−C1, N−C_2_), 1277 and 1292 cm^−1^)^42^ (Scheme 2e), two differences could be clearly observed for that of the other samples^43^. The carbonyl vibration band and hydrogen bonding band of PVP shifted to lower wavenumber (1659 to 1649 cm^−1^, 3452 to 3359 cm^−1^) when the Pd^2+^ and (or) Ti-O-Ti network^26^ adhesive incorporated into the PVP (Fig. 3b,c,d). PVP can form hydrogen bonding between terminal hydroxyl groups with carbonyl side-groups of repeat units in PVP macromolecules, and metal ions due to the structure of polyvinyl skeleton with strong polar group (pyrrolidone ring) with lone pair of electrons from the nitrogen and oxygen atoms^44^ (Fig. 3b), this implied that the strength of the dipole - dipole interactions of the PVP units was reduced. Here, PVP acted as a template and played a self-assembly role in the solution. After calcination and before reduction, PdO and TiO_2_ NPs were taken shape in the absence of PVP. Therefore, the porous composite nanofibers were formed.

### Gas Sensing Measurements

**Figure 4:**
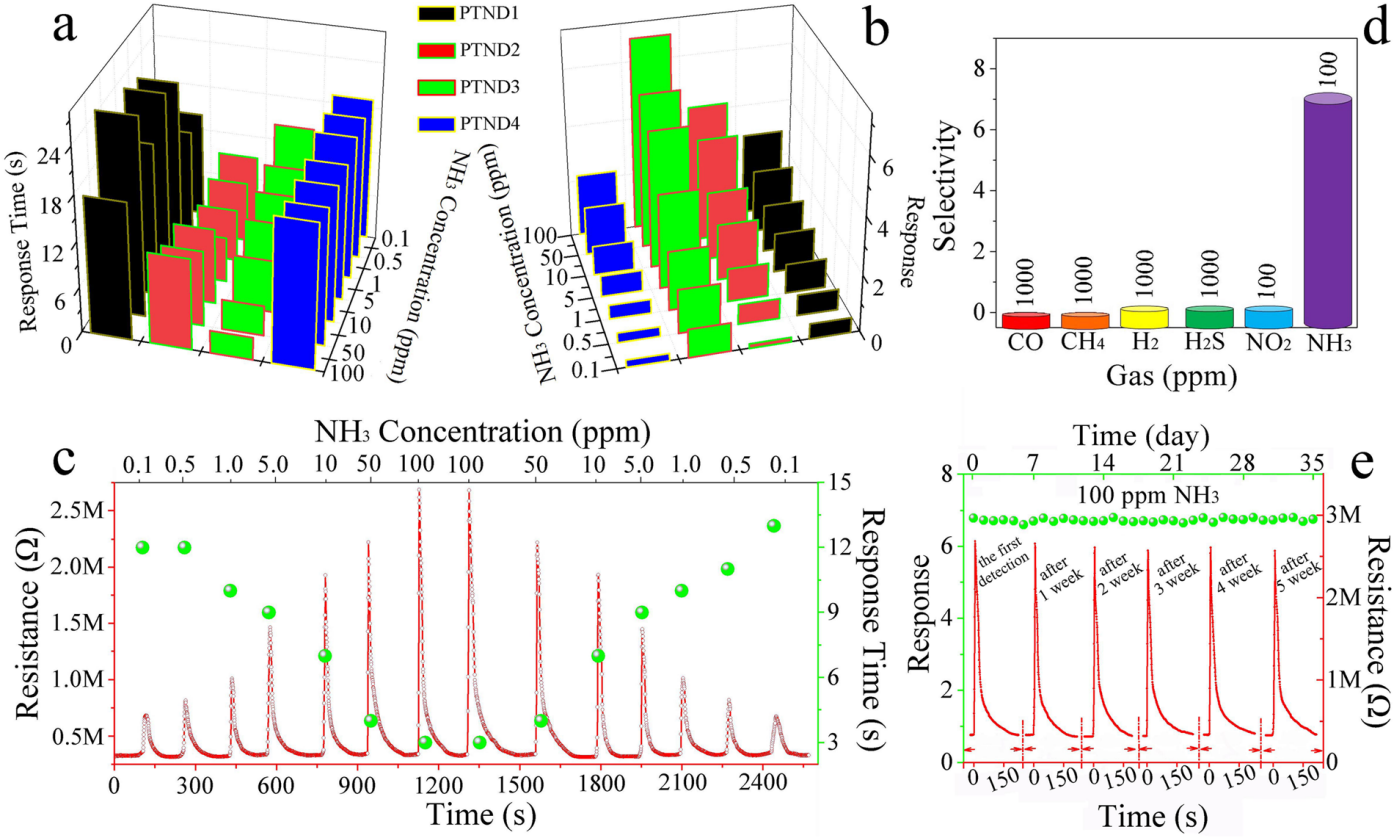
(**a**,**b**) The response time and gas response of samples to NH_3_, (**c**) Dynamic response of the PTND3 sensor to 0.1–100–0.1 ppm NH_3_ at 26 °C, the selectivity (**d**) and the stability (**e**) of PTND3 (inset in the top of (**e**) is Response/recovery curves of the sensor to 100 ppm NH_3_ at a certain interval) (RT: 26 °C, humidity of 30%).

Gas response and response time are the basic parameters of gas sensors, rapid response to a target gas are demanded for practical application. Therefore, gas sensing performances of the PTNDs NFs sensor and the pPTND3 sensor were evaluated and compared its performance with those of the pure TiO_2_ sensor to NH_3_ at 26 °C (fabricated process of the thin film gas sensor in Supporting Information and the SEM image of the sensor coated with the composite thin film in Fig. S6). The tests show that there is no response to pure TiO_2_ sensor to 100 ppm NH_3_ (in Fig. S7). Figure 4a,b show the response and response time of PTND1, PTND2, PTND3 and PTND4 sensors to NH_3_. (see Fig. 4c, Fig. S8 and Table S3). The responses of four sensors to NH_3_ tend to become stronger with increasing NH_3_ concentration. Generally, adding appropriate amount of noble metals (such as Pd) to gas-sensing material can effectively reduce response times^45,46^. Obviously, the PTND3 sensor exhibits higher response and quickly response time than other sensors (PTND1, PTND2, PTND4 and pPTND3 see Table S3).

Figure 4c illuminates the response/recovery properties and response time of the PTND3 sensor to 0.1–100–0.1 ppm NH_3_ at 26 °C (the dynamic response diagrams and corresponding data of PTND1, PTND2, PTND4 and pPTND3 sensors to different concentrations of NH_3_ are in Fig. S8, Table S3 and Table S4). Upon exposure to NH_3_, the resistance of PTND3 leads to a rapid increase, and after NH_3_ is removed, the resistance returns to the original value, which is the representative property of a p-type semiconductor. It is also observed that with the increase of NH_3_ concentration, the response of the PTND3 sensor is obviously increased, and the response time of the sensor is significantly reduced. The response time towards 100 ppm NH_3_ is about 3 s, and ∼14 s to 0.1 ppm NH_3_. Meantime, the response of the PTND3 to NH_3_ is 1 even at the concentration of 100 ppb. Not only that the PTND3 sensor shows a short-term reliability after two period tests, and an approximately linear response-concentration relationship in the range of 0.1–100 ppm NH_3_ to PTND3 sensor, as shows in Fig. 4c and in Fig. S9, which indicated that PTND3 has a potential application in combustible and noxious NH_3_ detection.

Since the selectivity and a long-term stability are also important parameters of gas sensor. The sensor must have rather high selectivity and stability for its application. Figure 4d depicts the selectivity of PTND3 sensor at 26 °C. The sensor shows high selectivity for NH_3_, and could distinguish an extremely small amount of 100 ppb NH_3_ compared with CO, CH_4_, H_2_, H_2_S, and NO_2_. The excellent selectivity further confirms that PTND3 sensor can be used as a promising material for NH_3_ sensor.

Figure 4e shows the response/recovery curves of the sensor to100 ppm NH_3_ at 26 °C within 35 days. The tests were carried out in the same interval (7 days) under the same conditions. The PTND3 sensor shows a stable gas response with 6.97 after repeated tests. It is found that the signal has no obvious changes to repeat many times, which illustrated illustrated superior stability of PTND3 sensor.

It is ture that humidity is prone to change significantly and rapidly in most environments. And the gas response of the PTND3 sensor had been tested under different humidity in order to measure its practicality at different environmental humidity. Fig. S10 shows the curves of the gas response for the PTND3 sensor to NH_3_ at 26 °C under different humidity. It is observed that with the increase of humidity from 20% to 60%, there is no obvious variation of the response of the PTND3 sensor, and the response of the sensor is significantly reduced when the humidity is increase to 70%. It illustrates that the PTND3 sensor has higher stability when the humidity is about 20%–60%.

In addition, there is no response for pure TiO_2_ sensor to 100 ppm NH_3_ (in Fig. S7), and the gas-sensing properties of PTND3 sensor (PTND3 was calcined under the mixed atmosphere of N_2_ and air, and it has 2% Pd) are better than pPTND3 sensor (pPTND3 was calcined under air, and it has 2% Pd too) (see Table S3 and Table S4). This demonstrated that, for PTND3 and pPTND3 sensor, the influenced factors for gas sensing properties was not just the electronic sensitization and chemical sensitization of Pd NPs^27,36,47–50^ in Pd@TiO_2_ NFs.

## Discussion

### The influence factors on gas sensing property

**Figure 5:**
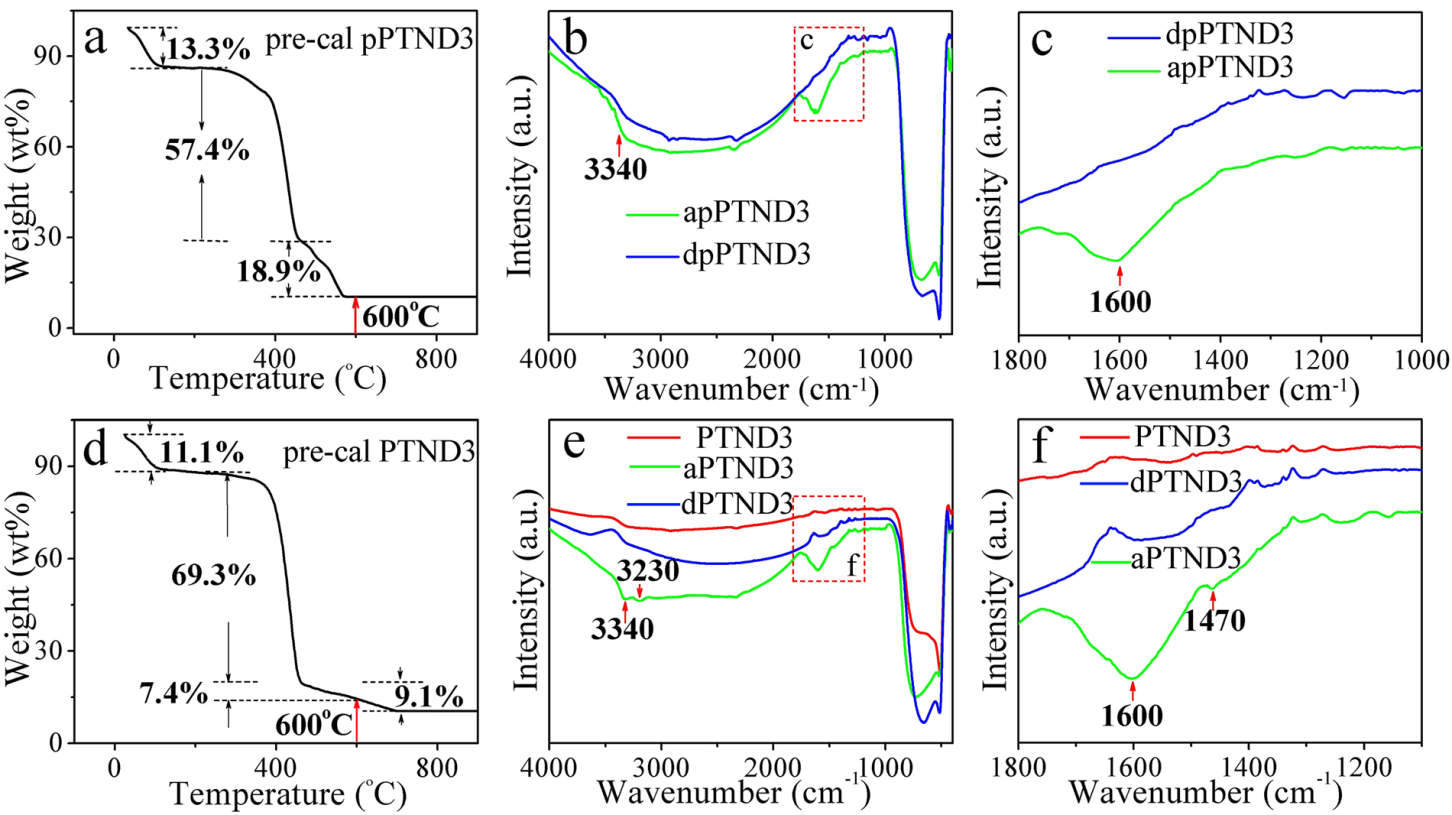
(**a**,**d**) TG curve of pre-cal pPTND3 and pre-cal PTND3; (**b**,**c**) IR spectra curves of apPTND3 (pPTND3 adsorbed NH_3_) and dpPTND3 (pPTND3 desorbed NH_3_); (**e**,**f**) IR spectra curves of PTND3, aPTND3 (PTND3 adsorbed NH_3_) and dPTND3 (aPTND3 desorbed NH_3_) (RT: 26 °C, humidity of 30%, synthesis and treatment conditions of above mentioned samples see in Table S1).

The elemental compositions of pPTND3 and PTND3 NFs are further researched because of calcining under the different atmosphere. Figure 5a and d describe the TG curves of pre-cal pPTND3 and pre-cal PTND3 NFs (TG treatment conditions and calcined products of above mentioned samples see in Table S2), respectively. TG curve of the pre-cal pPTND3 NFs was measured under air in the temperature range of 25–900 °C. Three stages of weight losses can be seen from Fig. 5a. The first stage of weight losses (ca. 13.3%) below 140 °C is mainly attributed to the loss of ethanol and water. The second loss stage (ca. 57.4%) from 270 °C to 460 °C is primarily due to the decomposition of PVP side chains. The third one (ca. 18.9%) in the range of 460–560 °C mostly results from the decomposition of main chain of PVP^51,52^. The total weight loss is about 89.6%. And at the same calcined condition, pure PVP was completely breakdown (as shown in Fig. S11). It can be roughly deduced that the 10.4% of the total weight of sample calcined is TiO_2_ and PdO.

TG curve of the pre-cal PTND3 NFs was measured under the mixture atmosphere of N_2_ and air in the temperature range of 25–900 °C. Three stages of weight losses in the range of 25–940 °C is about 89.5% (remaining 10.5% of TiO_2_ and PdO). The third one in the range of 490–940 °C chiefly results from the decomposition of main chain of PVP. Thus, there is some of C and N element (~16.2%) in remnant in PTND3 NFs after calcined at 600 °C under the N_2_ and air atmosphere and reduced (the TG curve of pure PVP calcined under the mixture atmosphere of N_2_ and air in Fig. S12).

IR and XPS spectra of samples (pure TiO_2_, PTND3 and pPTND3) adsorbed and desorbed NH_3_ were investigated in order to find the interactions between the samples and NH_3_ at 26 °C.

After pPTND3 adsorbed NH_3_ at 26 °C (in Fig. 5b,c) compare with pure TiO_2_ and apure TiO_2_ (see Fig. S13), it can be observed that, besides the peak of Ti-O, its IR spectra displays additional features corresponding to two distinct NH_3_ species: bands at 3340 and 1600 cm^−1^, they are assigned to adsorb NH_3_ on Lewis acid sites^53,54^.

In Fig. 5e,f, the IR spectra of PTND3 not only display bands at 3340 and 1600 cm^−1^ which are assigned to adsorb NH_3_ on Lewis acid sites, but also have bands at 3230 and 1470 cm^−1^ (see Fig. 5f) which are attributed to adsorb NH_3_ on Brønsted acid sites^55^. That means, both Lewis acid sites and Brønsted acid sites are existing in PTND3. This demonstrates that the synergy of Pd NPs and 16.2% of elements of C, N in PTND3 might play an important role in the generation of acid sites. Furthermore, to the curve of dPTND3 (Fig. 5f), NH_3_ species are disappearance basically.

**Figure 6:**
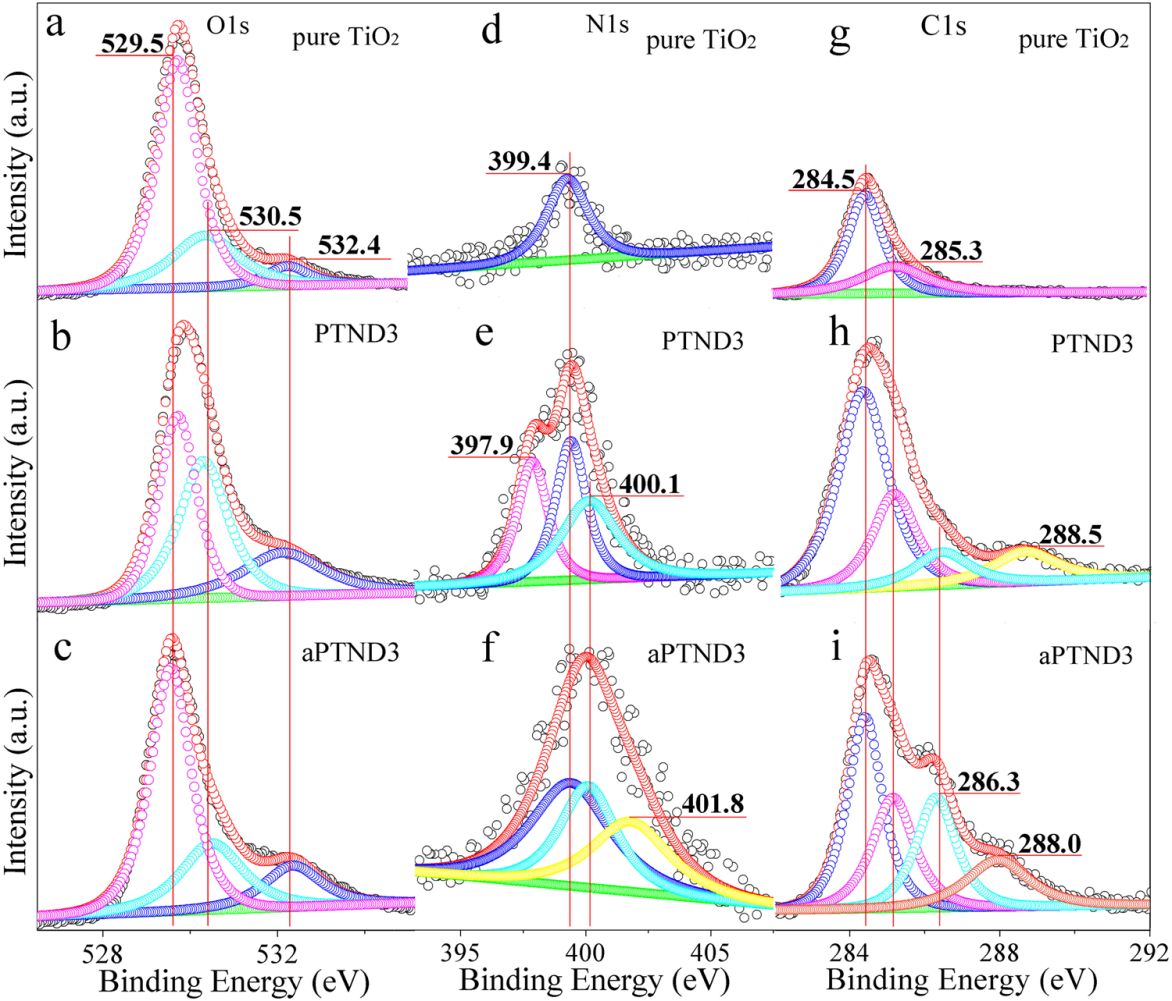
Curve-fitted XPS spectra of O 1 s (**g**–**i**), N 1 s (**j**–**l**) and C 1 s (**m**–**o**) of pure TiO_2_, PTND3 and aPTND3 (RT: 26 °C, humidity of 30%, synthesis and treatment conditions of above mentioned samples see Table S1).

In order to further research the effect of O, C and N element on the property of Pd@TiO_2_ NFs, XPS measurements were applied. As shown in Fig. 6a,b,c and in Fig. S14a,b, for pure TiO_2_, PTND3, aPTND3, pPTND3, and apPTND3, the O 1 s spectrum can be recounted into three peaks: 529.5, 530.5 and 532.4 eV, which are attributed to lattice oxygen, oxygen defect and adsorbed oxygen. The oxygen defect of apPTND3, aPTND3, pure TiO_2_, pPTND3 and PTND3 are 24.7%, 25.0%, 25.6%, 37.4% and 39.8%, respectively. The increased contribution percentage of oxygen defect in PTND3 NFs and pPTND3 NFs can be considered that the effect of deformation of introducing Pd NPs and the remains of elements of C, N in TiO_2_ might enhance the oxygen defect on the surface of TiO_2_ grains. Since more the oxygen defects, more will be the adsorption of gas^25^. In the meantime, the oxygen defect might directly or indirectly promote formation of Lewis acid sites or Brønsted acid sites, and improve their gas sensing performance.

As shown in Fig. 6d,e,f, N (1 s) in the samples which calcinated under the mixture atmosphere of N_2_ and air was studied by means of XPS spectrum. The N (1 s) XPS spectrum of pure TiO_2_ has only a little peak at 399.4 eV which is amino generally considered in non-reactive molecules. The N element which exists on the surface of PTND3 is amino iminic nitrogens -N = (397.9 eV), amino (399.4 eV) and -NH- (400.1 eV) states (see Fig. 6e). After NH_3_ adsorption, the weak NH_4_^+^ (401.8 eV) peak could be observed in aPTND3 (see Fig. 6f), expect amino (399.4 eV) and -NH- (400.1 eV) peaks^56–59^. It is consistent with the result of IR results, namely that Brønsted acid sites of PTND3 were formed because of existence of Pd NPs and elements of C and N, compared with pPTND3 and pure TiO_2_ (pPTND3, which was oxidized throughly, has no elements of N, and pure TiO_2_ has no elements of Pd). And ammonia can be adsorbed on the Brønsted acid sites forming NH_4_^+^ species and then the ability of adsorption NH_3_ will increase. Therefore, the response of PTND3 sensor enhanced compared with pPTND3 sensor.

In addition, the C 1 s XPS spectrum of pure TiO_2_ can be deconveluted into two peaks at 284.5 eV and 285.3 eV (Fig. 6g), which are attributed to the typical signals of C=C (sp^2^) and C-C (sp^3^), and PTND3 and pPTND3 are revealed from the C=C (sp^2^) at 284.5 eV, C-C (sp^3^) at 285.3 eV, C-O at 286.3 eV and COO at 288.5 eV (in Fig. 6h and Fig. S15a). Meanwhile, there are clearly exhibited C=C (sp^2^), C-C (sp^3^), C-O and C-NH_x_ components in the sample of aPTND3 and apPTND3, which located at 284.5, 285.3, 286.3, and 288.0 eV^54,60,61^, respectively (in Fig. 6i and Fig. S15b). The C-NH_x_ band located at 288.0 eV may be assigned to the adsorbed NH_3_ on the acid sites which formed on the surface of PTND3 and pPTND3 and resulted from synergy effect between Pd and C (or N) element. And this means that the response of PTND3 (in particular) and pPTND3 to NH_3_ was promoted up compared with pure TiO_2_.

Temperature-programmed desorption (TPD) is a surface science technique which has been used extensively to study surface reactions and molecular adsorption and desorption^62^. NH_3_-TPD were carried out to further investigate the acid strength of samples. Fig. S16 depicts the NH_3_-TPD patterns of both pure TiO_2_, PTND1, PTND2, PTND3, PTND4 and pPTND3. As shown in Fig. S16, the NH_3_ desorption peaks of pure TiO_2_ are too weak to be clearly observed, which suggested that pure TiO_2_ is shortage of acid sites. However for all of the Pd@TiO_2_, a larger peak was observed from 110 °C to approximately 450 °C compared with pure TiO_2_. Thus, it seemed that there was a broad distribution of weak and moderate acidity in the samples which contains Pd. As refer to the IR and XPS results (Figs 5 and 6), this implies that the peaks of Pd@TiO_2_ could be explained that the acidic sites were generated due to interacting between TiO_2_ and Pd. Furthermore, the increasing area of desorption peaks indicates that the acid strength is improved with the increasing of Pd amount, but the difference is not significant for Pd (2.0%) and Pd (2.5%). Meantime, there was weaker desorption peak at low temperature in the pPTND than that of PTND because of lackage of Brønsted acid sites of pPTND^63^. Therefore, the strong acidity decreased as the following order: PTND1 > PTND2 ≈ PTND3 > PTND4 > pPTND3 > TiO_2_.

### Gas sensing mechanism

The NH_3_ sensing properties of the PTND3 sensor have been significantly improved compared with pure TiO_2_ and pPTND3 depend upon the presence and interaction with Pd NPs, N and C elements in TiO_2_ NFs. And the gas-sensing mechanism of PTND3 sensor was probed into on the relationship between its structure and properties.

Firstly, a pure TiO_2_ (600 °C for 4 h under the mixed atmosphere of N_2_ and air) sensor could generate oxygen ions (O_2_^−^) when it is placed in an open atmosphere at RT. Nevertheless, the tests showed that there was no response to pure TiO_2_ sensor to 100 ppm NH_3_ (in Supporting Information, Fig. S7). This illustrates that it is hard to directly interact between NH_3_ molecules and oxygen ions (O_2_^−^), and there is no obviously effect between TiO_2_ and remaining C (or N) element.

And, secondly, pPTND3 (Pd 2.0%, 600 °C for 4 h under air atmosphere) sensor could produce a large number of oxygen ions (O_2_^−^), when it is placed in an open atmosphere at RT. As we known, the Pd NPs on the surface of metal oxide material can form electronic sensitization mechanism, that is, oxygen molecules can rapidly dissociate and forming oxygen ions (O_2_^−^) because of the catalyticaction of Pd NPs^27,31,33,34,36^. Not only that, the response of the pPTND3 sensor to the NH_3_ is 1.43 at the concentration of 100 ppm, it illustrates that interaction between NH_3_ molecules and oxygen ions happened with the presence of Pd. But at the same time, pPTND3 and PTND3 have identical Pd and oxygen ions (O_2_^−^) content but different NH_3_ response (the response of the PTND3 sensor to 100 ppm NH_3_ is 6.97), it explains that it is hard to directly interact between NH_3_ molecules and oxygen ions (O_2_^−^), in addition, remaining C and N elements in PTND3 not contained in pPTND3 also play an active role on the performance of gas sensing in addition to Pd. In the meantime, from IR spectrum, pPTND3 displays bands at 3340 and 1600 cm^−1^ which are Lewis acid sites which is easily to adsorb NH_3_ molecules, and PTND3 also has bands at 3230 and 1470 cm^−1^ (see Fig. 5f) which are Brønsted acid sites which can attribute to adsorb NH_3_ too. Thereby, it deduces that Pd NPs and TiO_2_ NFs could form Lewis acid sites and the interaction between Pd NPs, N and C elements in TiO_2_ NFs contributes to forming of Brønsted acid sites. So far, the gas-sensing mechanism of PTND3 sensor is investigated.

**Figure 7:**
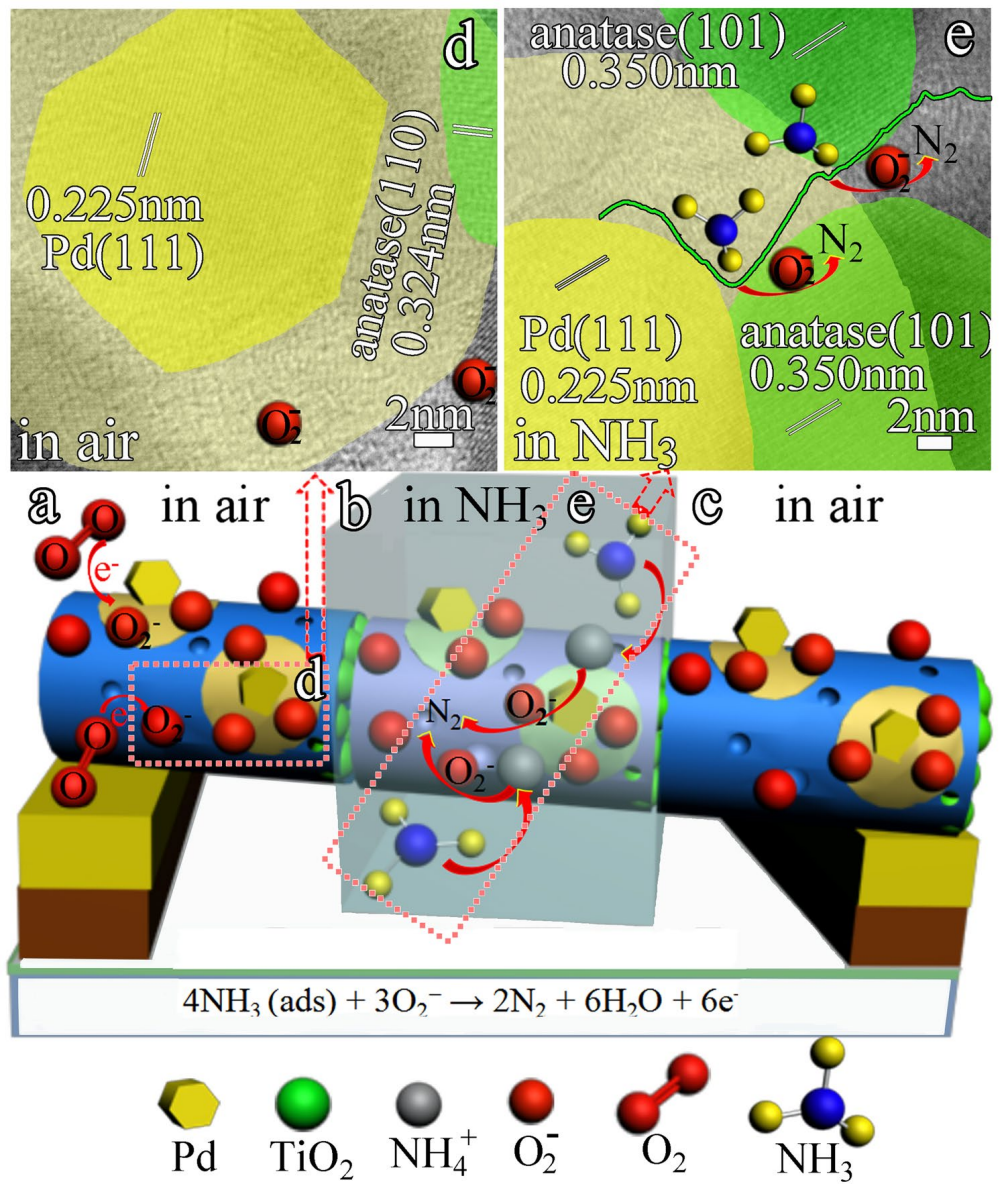
Mechanism of the PTND3 sensor upon exposure to air and NH_3_. (**a**) PTND3 sensor in air, (**b**) PTND3 sensor exposure to NH_3_, (**c**) PTND3 sensor recover in air.

Initially, as shown in Fig. 7a,d, when a PTND3 sensor is placed in an open atmosphere at RT, oxygen moleculars capture electrons from the surface of TiO_2_ NPs of nanofibers forming oxygen ions (O_2_^−^), and the Pd NPs catalytically activate oxygen moleculars into oxygen ions (O_2_^−^) (electronic sensitization mechanism), subsequently oxygen ions (O_2_^−^) diffuse into the TiO_2_ NPs of nanofibers around Pd NPs and form an activity region. This process greatly increases the quantity of oxygen ions (O_2_^−^) compared with pristine TiO_2_ NFs. Meanwhile, the interaction of Pd NPs, C and N elements in TiO_2_ NPs of nanofibers could form Lewis acid sites and Brønsted acid sites in the activity region around Pd NPs.

On exposure to NH_3_ at RT (Fig. 7b,e), NH_3_ molecules can be easily adsorbed on Lewis acidic sites and Brønsted acidic sites of the activity region on PTND3. Under the impact of chemical sensitization mechanism of Pd NPs, oxygen ions (O_2_^−^) can promptly interact with NH_3_ and readily release more electrons back to conduction band of TiO_2_ NPs of nanofibers (see reactions 2), which leading to an decrease in the carrier concentration of p-type TiO_2_ NPs active layer. Hence the resistance of the PTND3 sensor swiftly increases.

Equations (1–2) are the corresponding reactions^64,65^.

1 $${{\rm{O}}}_{{\rm{2}}}({{\rm{TiO}}}_{{\rm{2}}}{\rm{adsorbed}})+{{\rm{e}}}^{-}\to {{{\rm{O}}}_{2}}^{-}$$

2 $${{\rm{4NH}}}_{{\rm{3}}}({\rm{ads}})+3{{{\rm{O}}}_{2}}^{-}\to 2{{\rm{N}}}_{2}+6{{\rm{H}}}_{2}{\rm{O}}+6{{\rm{e}}}^{-}$$

## Conclusion

The present study has demonstrated a one-step method to fabricate ordered porous Pd@TiO_2_ NFs array film. In particularly, PTND3 ordered nanofibers show a high sensing response and fast response time towards different concentration NH_3_. Most importantly, the PTND3 ordered nanofibers sensor present superior stability to 100 ppm after used 35 days.

The enhanced gas sensing could be ascribed to two factors (i) The electronic sensitization mechanism and chemical sensitization mechanism of Pd NPs on the TiO_2_ nanofibers, which could be more favorable to produce oxygen ions and accelerated the molecule-ion conversion rate, enhanced the reaction activity of oxygen ions (O_2_^−^) with NH_3_ compared with pristine TiO_2_ NFs; (ii) The synergistic effect between Pd NPs, C and N element in TiO_2_ NFs could form Lewis acid sites and Brønsted acid sites which could enhance NH_3_ adsorption-desorption ability and activity, and thus favour the rapid-response between NH_3_ and oxygen ions. Therefore, the Pd@TiO_2_ NFs sensor exhibited excellent gas-sensing property at RT. Pd@TiO_2_ NFs array film would be effectively used in commercial gas-sensing and environmental monitoring applications.

## Experimental Techniques

### Sample preparation

All chemicals used were of analytical grade without further purification. The Pd@TiO_2_ NFs were synthesized by calcinated and reduced using NaBH_4_ aqueous solution. In a typical process, 7.2 g poly (N-vinylpyrrolidone) (*M*_W_ = 58 000) was dissolved in 15 mL ethanol, then 1 mL 0.005 mol/L H_2_PdCl_4_ solution and a certain amount of tetra butyl titanate (TBT, C_16_H_36_O_4_Ti, *M*_W_ = 340.32) was added to obtain a transparent precursor solution of different quality percentage Pd. The quality percentage of Pd is 3.0, 2.5, 2.0 and 1.5 wt%, respectively. The mixed solution was stirred vigorously at RT for 5 h and then transferred into a 10 mL syringe with a capillary tip (0.8 mm in diameter) for spinning. A high voltage of 18 kV was applied at the spinneret as a direct-current power supply (BGG, DFS-01, Beijing Kaiweixin Technique Co., China), and the nanofibers were collected using the aluminium foil at RT. Then the nanofibers blankets were taken off the aluminium foil, and treated at 600 °C for keeping 4 h under the mixed atmosphere of N_2_ and air (in a proportion of 1 to 1) with a heating rate of 3 °C min^−1^. The nanofibers array film was obtained. And then, 0.05 g nanofibers array film was added into 20 mL 0.01 mol/L NaBH_4_ aqueous solution at one time. The color of solution changed from yellow to dark-brown gradually. The reduction reaction lasted for 10 h under impregnation. Subsequently the sample was filtered with washing by distilled water, and dried at 60 °C for 10 h. Pd@TiO_2_ NFs with 3.0, 2.5, 2.0 and 1.5 wt% of Pd were obtained and marked as PTND1, PTND2, PTND3, and PTND4, respectively. Here, pPTND3 which the only difference from PTND3 was calcined under air atmosphere. In addition, apure TiO_2_ was the sample which pure TiO_2_ adsorbed 100 ppm NH_3_ at RT for 30 min, aPTND3 was the sample which PTND3 adsorbed 100 ppm NH_3_ at RT for 30 min, and dPTND3 was the one when aPTND3 desorbed NH_3_ from RT to 600 °C under N_2_ with a heating rate of 5 °C min^−1^. Moreover, in order to compare the effects after the different samples adsorbed or desorbed NH_3_, apPTND3 (pPTND3 adsorbed 100 ppm NH_3_ from RT to 600 °C under N_2_ with a heating rate of 5 °C min^−1^ as the same condition of aPTND3) and dpPTND3 (apPTND3 desorbed NH_3_ from RT to 600 °C under N_2_ with a heating rate of 5 °C min^−1^ as the same condition of dPTND3) were prepared.

### Characterization

The crystal phase of samples was characterized by X-ray powder diffraction (XRD, D/MAX-Ш-B-40 KV, Japan, Cu-Kα radiation, λ = 1.5406 Å). The morphology and structures of the nanofibers were observed by scanning electron microscope (SEM, HITACHI S-4800) with an acceleration voltage of 5 kV, and transmission electron microscopy (TEM, JEOL-2100) with an acceleration voltage of 200 kV. The Brunauer-Emmett-Teller (BET) surface area of the products was measured by N_2_ adsorption-desorption (TriStar II 3020). The sample was dried for 10 h at 150 °C under vacuum before the measurement. X-Ray photoelectron spectra (XPS) analyse was performed with a VGESCALAB MK II using MgKα (1253.6 eV) achromatic X-ray radiation under high vacuum of 5 × 10^−7^ Pa. Infrared spectra (IR) were recorded on a Nicolet Antaris Fourier transform spectrometer between 400 and 4000 cm^−1^. Spectra were obtained after 20 accumulations with a resolution of 4 cm^−1^. Thermogravimetric (TG) curves were obtained with a Shimadzu TGA-50 using a platinum crucible with a sample mass of 10 mg. The heating rate was 10 °C min^−1^, between 25 and 1200 °C, in air atmosphere or mixture atmosphere of air and N_2_ (50 mL · min^−1^). Temperature-programmed desorption (TPD) experiments were carried out with a TP-5000 multi-purpose adsorption instrument using Chemisorp 2705 unit (Micromeritics Instrument. Co., USA) equipped with thermal conductivity detector (TCD) to detect NH_3_ adsorption and desorption behavior.

### Gas sensing test

The fabrication and measurements of gas sensors were made from the as-synthesized composites by the method we reported before^25^. An alumina substrate (7 mm × 5 mm × 0.38 mm, content 99.6%) with two interdigitated Au electrodes on its top surface was used. Each Au electrode contained 50 fingers, and the distance between two fingers was 20 µm. The Au electrodes on Al_2_O_3_ substrate was cleaned by diluted HCl, distilled water and acetone. Nanofibers array film was placed on the Au electrodes under the microscope, then calcined at 350 °C for 1 h in N_2_ and air (or air) to obtain nanofibers array film sensor.

The sensor was installed inside a test chamber with an inlet and an outlet in. The electrical resistance measurements of the sensor was carried out at RT (26 °C) and the relative humidity (RH) around 30%. The gas concentration was controlled by injecting a volume of target gas into the chamber in a certain amount of time with a small fan, and then the chamber was purged with air to revert to the original baseline. The resistance change of the thin film sensor has been measured to confirm the interactions of the sensor and the NH_3_ gas. The gas response was defined as the ratio |R_g_ − R_a_|/R_a_, where R_a_ and R_g_ are thin film resistance, measured in air atmosphere and NH_3_ gas, respectively. The response time is defined as the time taken for the sensor to attain 90% of the maximum change in resistance on exposure to the target gas.

### TPD analysis

Typically, the sample of 50 mg loaded in the quartz tube was firstly pretreated with He of 30 mL·min^−1^ at 250 °C for 0.5 h, and subsequently the sample was saturated with NH_3_(5% in He) in a flow rate of 30 ml·min^−1^ for about 30 min at 100 °C. After that, the physically adsorbed NH_3_ was purged by high purity He at the same temperature. The chemisorbed NH_3_ was measured by heating the sample in He (30 ml·min^−1^ from 100 °C to 600 °C with a heating rate of 10 °C·min^−1^).

### Data availability statement

The authors declare that the data in the manuscript are true and reliable.

## Electronic supplementary material

Supplementary Information

## References

[1] Poulomi R, Steffen B, Patrik S (2011). TiO_2_ Nanotubes: Synthesis and Applications. Angew. Chem..

[2] Wei W (2017). Single-step One-pot Synthesis of Graphene Foam/TiO_2_ Nanosheet Hybrids for Effective Water Treatment. Sci. Rep..

[3] Ting Z, Wei O, Liang Z, Ghim W (2015). TiO_2_ Fibers Supported β-FeOOH Nanostructures as Efficient Visible Light Photocatalyst and Room Temperature Sensor. Sci. Rep..

[4] Meidan Y (2012). High-Efficiency Photoelectrocatalytic Hydrogen Generation Enabled by Palladium Quantum Dots-Sensitized TiO_2_ Nanotube Arrays. J. Am. Chem. Soc..

[5] Yuan W (2016). A novel ethanol gas sensor based on TiO_2_/Ag_0.35_V_2_O_5_ branched nanoheterostructures. Sci. Rep..

[6] Hao FL (2008). Amorphous TiO_2_ nanotube arrays for low-temperature oxygen sensors. Nanotechnology.

[7] Shi WL (2011). A selective room temperature formaldehyde gas sensor using TiO_2_ nanotube arrays. Sensors and Actuators B: Chem..

[8] Wang L (2006). Sensing arrays constructed from nanoparticle thin films and interdigitated microelectrodes. Sensors.

[9] Mei W, Dao-jun G, Hu-lin L (2005). High activity of novel Pd/TiO_2_ nanotube catalysts for methanol electro-oxidation. Journal of Solid State Chemistry.

[10] Karumbaiah NC, York RS, Mano M, Swomitra KM (2012). Site-specific and patterned growth of TiO_2_ nanotube arrays from e-beam evaporated thin titanium film on Si wafer. Nanotechnology.

[11] Karumbaiah NC (2015). Effect of Sputtering Parameters on the Morphology of TiO_2_ Nanotubes Synthesized From Thin Ti Film on Si Substrate. IEEE Transactions on Nanotechnology.

[12] Karumbaiah NC (2014). TiO_2_–WO_3_ Composite Nanotubes from Co–Sputtered Thin Films on Si Substrate for Enhanced Photoelectrochemical Water Splitting. J. Electrochem. Soc..

[13] Karumbaiah NC, Mano M, Swomitra KM (2017). Synthesis of TiO_2_–Al_2_O_3_ and TiO_2_–Ta_2_O_5_*composite nanotubes on* planar Si substrates. Micro & Nano Letters.

[14] Karumbaiah NC (2012). Growth and characterization of TiO_2_ nanotubes from sputtered Ti film on Si substrate. Nano. Res. Lett..

[15] York RS, Karumbaiah NC, Swomitra KM, Mano M (2014). TiO_2_-WO_3_ Nanotubular Composite Synthesized by Anodization of Simultaneous Multi-Target Sputtered Thin Films Characterized by Laser Ablation ICP-MS. ECS Trans..

[16] Xiao XW, Guang FH, Hao F, Zheng TZ (2013). Electron Transport and Recombination in Photoanode of Electrospun TiO_2_ Nanotubes for Dye-Sensitized Solar Cells. J. Phys. Chem. C.

[17] Bin GL (2012). Preparation of highly porous TiO_2_ nanotubes and their catalytic applications. J. Mater. Chem..

[18] Ignacio GL (2004). Electrically Forced Coaxial Nanojets for One-Step Hollow Nanofiber Design. J. Am. Chem. Soc..

[19] Marasso SL (2016). A new method to integrate ZnO nano- tetrapods on MEMS micro-hotplates for large scale gas sensor production. Nanotechnology.

[20] Ivanov P, Stankova M, Llobet E (2005). Nanoparticle metal-oxide films for micro-hotplate-based gas sensor systems. IEEE Sens..

[21] Francioso L (2008). Linear temperature microhotplate gas sensor array for automotive cabin air quality monitoring. Sensors Actuators, B Chem..

[22] Bak T, Nowotny J, Nowotny MK (2006). Defect Disorder of Titanium Dioxide. J. Phys. Chem. B..

[23] Nowotny J, Radecka M, Rekas M (1997). Semiconducting properties of undoped TiO_2_. J. Phys. Chem. Solids..

[24] Nowotny MK, Sheppard LR, Bak T, Nowotny J (2008). Defect Chemistry of Titanium Dioxide. Application of Defect Engineering in Processing of TiO_2_-Based Photocatalysts. J. Phys. Chem. C..

[25] Wu HY (2014). Electrospinning of mesoporous p-type In_2_O_3_/TiO_2_ composite nanofibers for enhancing NO_x_ gas sensing properties at room temperature. CrystEngComm..

[26] Wu HY (2016). Facile preparation of porous In_2_TiO_5_-rutile composite nanotubesby electrospinning and sensitivity enhancement in NO_2_ gas at room temperature. J. Colloid Interface Sci..

[27] Mohit K (2017). Pd/ZnO nanorods based sensor for highly selective detection of extremely low concentration hydrogen. Sci. Rep..

[28] Park S, Kim S, Sun GJ, Lee C (2015). Synthesis, Structure, and Ethanol Gas Sensing Properties of I_n_2_O_3 Nanorods Decorated with B_i_2_O_3 Nanoparticles. ACS Appl. Mater. Interfaces.

[29] Long H (2016). *In Situ* Localized Growth of Porous Tin Oxide Films on Low Power Microheater Platform for Low Temperature CO Detection. ACS Sens..

[30] Tianyu L (2016). Scalable Synthesis of Ag Networks with Optimized Sub-monolayer Au-Pd Nanoparticle Covering for Highly Enhanced SERS Detection and Catalysis. Sci. Rep..

[31] Wei W, Fu Y, Xiao N, Yu Z (2016). Preparation of Pd supported on La(Sr)-Mn-O Perovskite by microwave Irradiation Method and Its Catalytic Performances for the MethaneCombustion. Sci. Rep..

[32] Liu X (2010). Amino acid-assisted one-pot assembly of Au, Pt nanoparticles onto one-dimensional ZnO microrods. Nanoscale.

[33] Dong H (2015). Flower-like Palladium Nanoclusters Decorated Graphene Electrodes for Ultrasensitive and Flexible Hydrogen Gas Sensing. Sci. Rep..

[34] Yu G, Wu S, Wei Y, Qi L (2017). Creation of Pd/Al_2_O_3_ Catalyst by a Spray Process for Fixed Bed Reactors and Its Effective Removal of Aqueous Bromate. Sci. Rep..

[35] Dachi Y (2014). Shape-controlled synthesis of palladium and copper superlattice nanowires for high-stability hydrogen sensors. Sci. Rep..

[36] Lin Y (2014). Room-temperature self-powered ethanol sensing of a Pd/ZnO nanoarray nanogenerator driven by human finger movement. Nanoscale.

[37] Yi J (2015). AgI/TiO_2_ nanobelts monolithic catalyst with enhanced visible light photocatalytic activity. J. Hazard. Mater..

[38] Gabal MA, Hameed SA, Obaid AY (2012). CoTiO_3_ via cobalt oxalate-TiO_2_ precursor. Synthesis and characterization. Mater. Charact..

[39] Sing KSW (1985). Reporting physisorption data for gas/solid systems with special reference to the determination of surface area and porosity. Pure and Appl. Chem..

[40] Yohan P, Seog KK, Debabrata P, Youngku S (2014). Thermal H_2_-treatment effects on CO/CO_2_ conversion over Pd-doped CeO_2_ comparison with Au and Ag-doped CeO_2_. Reaction Kinetics, Mechanisms and Catalysis.

[41] Xiang Y, Meng Q, Li X, Wang J (2010). *In situ* hydrogen from aqueous-methanol for nitroarene reduction and imine formation over an Au-Pd/Al_2_O_3_ catalyst. Chem. Commun..

[42] Xian J (2012). Size-Dependent Interaction of the Poly(N-vinyl-2-pyrrolidone) Capping Ligand with Pd Nanocrystals. Langmuir.

[43] Taghizadeh SM, Mirzadeh H, Barikani M, Yousefi M (2009). Miscibility and tack of blends of poly (vinylpyrrolidone)/acrylic pressure-sensitive adhesive. Int. J. Adhes. Adhes..

[44] Zhu, J. *et al*. High-Yield Synthesis of Uniform Ag Nanowires with High Aspect Ratios by Introducing the Long-Chain PVP in an Improved PolyolProcess. *Nanomater*. 982547–982554 (2011).

[45] Hyunsu K (2012). H_2_S gas sensing properties of bare and Pd-functionalized CuO nanorods. Sensors and Actuators B: Chemical.

[46] Jun ML (2010). Ultra-sensitive hydrogen gas sensors based on Pd-decorated tin dioxide nanostructures: Room temperature operating sensors. International Journal of Hydrogen Energy.

[47] Kolmakov A (2005). Enhanced Gas Sensing by Individual SnO_2_ Nanowires and Nanobelts Functionalized with Pd Catalyst Particles. Nano Lett..

[48] Wang L (2013). A Au-functionalized ZnO nanowire gas sensor for detection of benzene and toluene. Phys. Chem. Chem. Phys..

[49] Hwang I (2011). Facile Control of C_2_H_5_OH Sensing Characteristics by Decorating Discrete Ag Nanoclusters on SnO_2_ NanowireNetworks. ACS Appl. Mater. Interfaces.

[50] Hübner M (2012). An Au clusters related spill-over sensitization mechanism in SnO_2_-based gas sensors identified by operando HERFD-XAS, work function changes, DC resistance and catalytic conversion studies. Phys. Chem. Chem. Phys..

[51] Wu J (2014). Mechanistic Insights into Formation of SnO_2_ Nanotubes: Asynchronous Decomposition of Poly (vinylpyrrolidone) in Electrospun Fibers during Calcining Process. Langmuir.

[52] Hagen R (2013). Influence of electrode design on the electrochemical performance of Li_3_V_2_(PO_4_)_3_/C nanocomposite cathode in lithium ion batteries. Nano Energy.

[53] Breysse M (2001). New aspects of catalytic functions on sulfide catalysts. Catal. Today.

[54] Cha W, Yun S, Jurng J (2014). Examination of surface phenomena of V_2_O_5_ loaded on new nanostructured TiO_2_ prepared by chemical vapor condensation for enhanced NH_3_-based selective catalytic reduction (SCR) at low temperatures. Phys. Chem. Chem. Phys..

[55] Zaki MI, Hasan MA, Al-Sagheer FA, Pasupulety L (2001). *In situ* FTIR spectra of pyridine adsorbed on SiO_2_-Al_2_O_3_, TiO_2_, ZrO_2_ and CeO_2_: general considerations for the identification of acid sites on surfaces of finely divided metal Oxides. Colloid Surf. A.

[56] Kim J (2007). Quantitative analysis of surface amine groups on plasma-polymerized ethylenediamine films using UV–visible spectroscopy compared to chemical derivatization with FT-IR spectroscopy, XPS and TOF-SIMS. Appl. Surf. Sci..

[57] Doyle CM (2011). Evidence for the formation of an intermediate complex in the direct metalation of tetra(4-bromophenyl)-porphyrin on the Cu(111) surface. Chem. Commun.

[58] Asthana H, Erickson BL, Drzal LT (1997). Sulfonation of polymer surfaces - II. Chemical changes on polypropylene and polystyrene surfaces after gas phase sulfonation. J. Adhes. Sci. Technol..

[59] Hersh LS, Onyiriuka EC, Hertl W (1995). Amine-reactive surface chemistry of zinc phosphate glasses. J. Mater. Res..

[60] Okpalugo TI (2005). High resolution XPS characterization of chemical functionalised MWCNTs and SWCNTs. Carbon.

[61] Yao C (2008). Surface modification and antibacterial activity of electrospun polyurethane fibrous membranes with quaternary ammonium moieties. J. Membr. Sci..

[62] Sara IN, Jeffrey WT (2006). Development of thermal programmed desorption mass spectrometry methods for environmental applications. Chemosphere.

[63] Cheng F (2013). Low-temperature selective catalytic reduction of NO with NH_3_ over nanoflaky MnO_x_ on carbon nanotubes *in situ* prepared via a chemical bath deposition route. Nanoscale.

[64] Xu S (2015). Enhanced NH_3_ gas sensing performance based on electrospun alkaline-earth metals composited SnO_2_ nanofibers. J. Alloys Compd..

[65] Gautam M, Jayatissa AH (2012). Ammonia gas sensing behavior of graphene surface decorated with gold nanoparticles. Solid-State Electron.

