# Supplementary material for: One-step Synthesis of Ordered Pd@TiO2 Nanofibers Array Film as Outstanding NH3 Gas Sensor at Room Temperature
Source: Sci Rep. 2017 Nov 7;7:14688. doi: 10.1038/s41598-017-15319-3 (PMC5677023; doi:10.1038/s41598-017-15319-3)
Supplement: Supplementary file 1 — Supplementary Information [file 41598_2017_15319_MOESM1_ESM.doc]

**One-step Synthesis of Ordered Pd@TiO_2_ Nanofibers Array Film as Outstanding NH_3_ Gas Sensor at Room Temperature**

**Hongyuan Wu^1,3^,** **Haitao Huang^4^, Jiao Zhou,^1^ Dahai Hong,^1^ Muhammad Ikram,^1^ Afrasiab Ur Rehman^1^, Li Li^1,2,*^ & Keying Shi^1,*^**

^1^Key Laboratory of Functional Inorganic Material Chemistry, Ministry of Education. School of Chemistry and Material Science, Heilongjiang University, Harbin, 150080, P.R. China

^2^Key Laboratory of Chemical Engineering Process＆Technology for High-efficiency Conversion，School of Chemistry and Material Science, Heilongjiang University, Harbin 150080, P. R. China

^3^College of Chemistry and Chemical Engineering, Qiqihar University, Qiqihar 161006, P.R. China

^4^The third affiliated hospital of Qiqihar Medical University, Qiqihar 161006, P.R. China

^*^Corresponding.[shikeying2008@163.com](mailto:shikeying2008@163.com); [llwjjhlju@sina.cn](mailto:llwjjhlju@sina.cn)

**Table S1.** Summary of synthesis and treatment conditions of samples

| Samples | Pd wt% | Treatment conditions:  600 ºC for 4 h  under different atmosphere | NH_3_  adsorption at room temperature (RT) | NH_3_  desorption at RT |
| --- | --- | --- | --- | --- |
| pure TiO_2_ | 0 | the mixed atmosphere of  N_2_ and air | - | - |
| apure TiO_2_ | 0 | the mixed atmosphere of  N_2_ and air | pure TiO_2_ adsorbed 100 ppm NH_3_ at RT for 30min | - |
| PTND1 | 3.0 | the mixed atmosphere of  N_2_ and air | - | - |
| PTND2 | 2.5 | the mixed atmosphere of  N_2_ and air | - | - |
| PTND3 | 2.0 | the mixed atmosphere of  N_2_ and air | - | - |
| PTND4 | 1.5 | the mixed atmosphere of  N_2_ and air | - | - |
| aPTND3 | 2.0 | the mixed atmosphere of  N_2_ and air | PTND3 adsorbed 100 ppm NH_3_ at RT for 30min | - |
| dPTND3 | 2.0 | the mixed atmosphere of  N_2_ and air | Firstly, PTND3 adsorbed 100 ppm NH_3_ as the same condition of aPTND3 | Secondly, aPTND3 desorbed NH_3_ from RT to 600 ºC under N_2_ with a heating rate of 5 ºC · min^-1^. |
| pPTND3 | 2.0 | atmosphere of air | - | - |
| apPTND3 | 2.0 | atmosphere of air | pPTND3 adsorbed 100 ppm NH_3_ as the same condition of aPTND3 | - |
| dpPTND3 | 2.0 | atmosphere of air | Firstly, pPTND3 adsorbed 100 ppm NH_3_ as the same condition of aPTND3 | Secondly, pPTND3 desorbed NH_3_ as the same condition of dPTND3 |

**Table S2.** Summary of synthesis and treatment conditions of samples

| Samples | Pd wt% | TG treatment conditions:  under different atmosphere | Calcined and reduced product |
| --- | --- | --- | --- |
| pre-cal PTND3 | 2.0 | the mixed atmosphere of  N_2_ and air | PTND3 |
| pre-cal pPTND3 | 2.0 | atmosphere of air | pPTND3 |

IR spectra were measured with using a Nicolet Antaris Fourier transform spectrometer at a resolution of 4 cm^-1^ and 100 scans/spectrum. Adsorption experiments were carried out in a stainless steel IR cell equipped with removable CaF_2_ windows. The sample was pressed into a self-supporting disc, ca. 15 mm in diameter. Pretreatment of the sample involved adsorpting 100 ppm NH_3_ for 30min at RT (26 ºC).

NH_3_-TPD was measured with a conventional TPD apparatus. Before adsorption, the sample was done from RT to 600 ºC under N_2_ with a heating rate of 5 ºC · min^-1^. Infrared spectra (IR) of the sample which has desorbed ammonia was also recorded on a Nicolet Antaris Fourier transform spectrometer between 400 and 4000 cm^-1^.


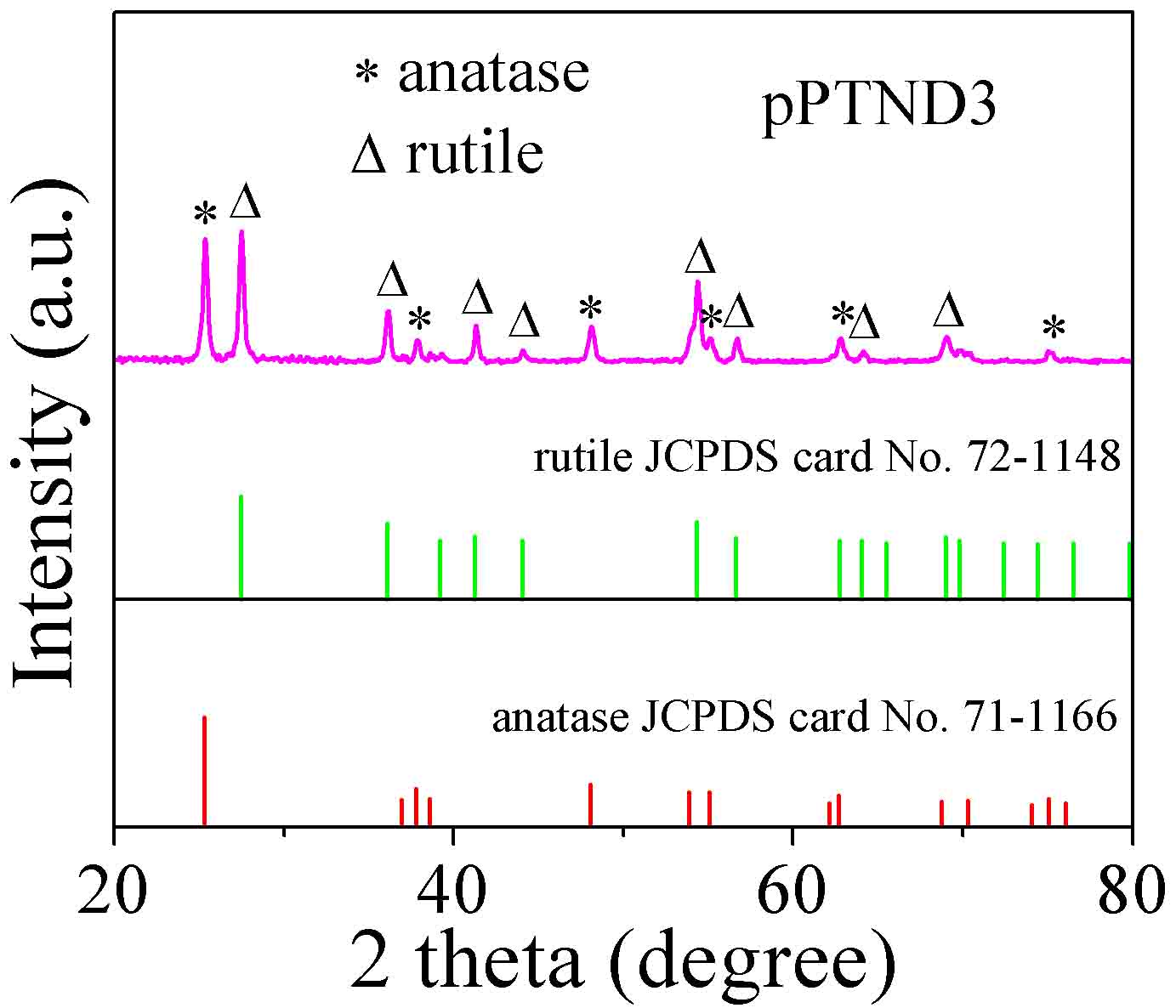


**Figure S1.** XRD pattern of pPTND3.


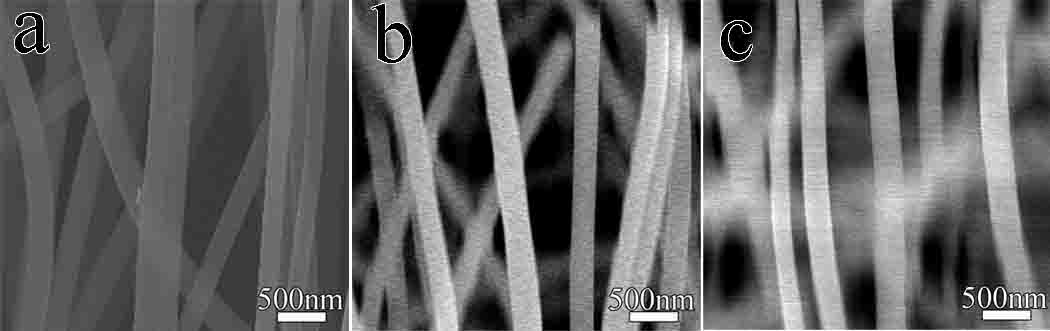


**Figure S2.** SEM images of (a) PTND1, (b) PTND2, (c) PTND4 nanofibers array.


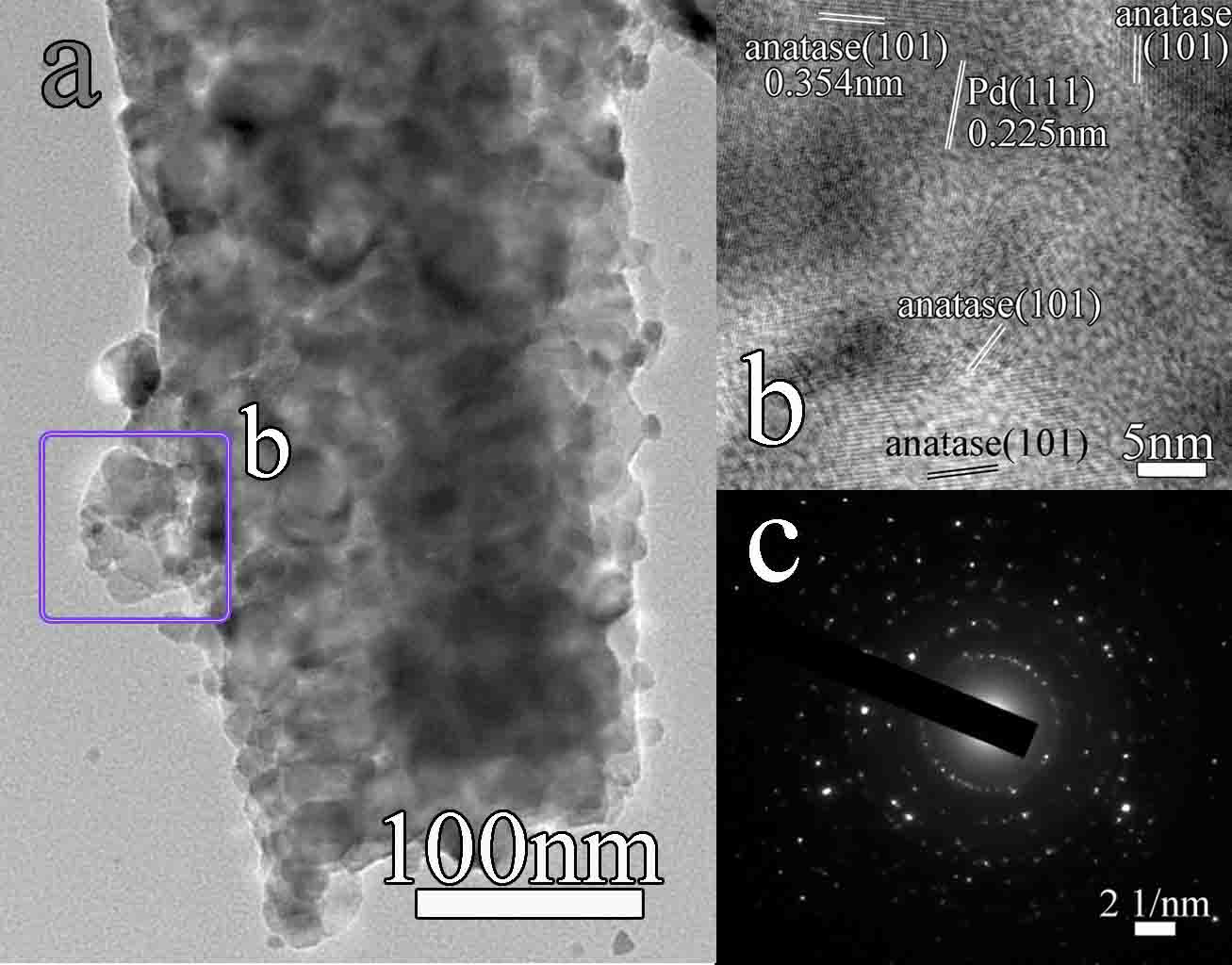


**Figure S3.** (a) TEM image of pPTND3 nanofibers after calcination, (b) HRTEM image with indicated d-spacings of pPTND3, (c) selected area electron diffraction (SAED) image of (a).

The corresponding selected area electron diffraction (SAED) pattern shown in Fig. S3 confirms highly crystalline nature of anatase (101), (004) and rutile (101), (110) planes, which is in good agreement with XRD results.


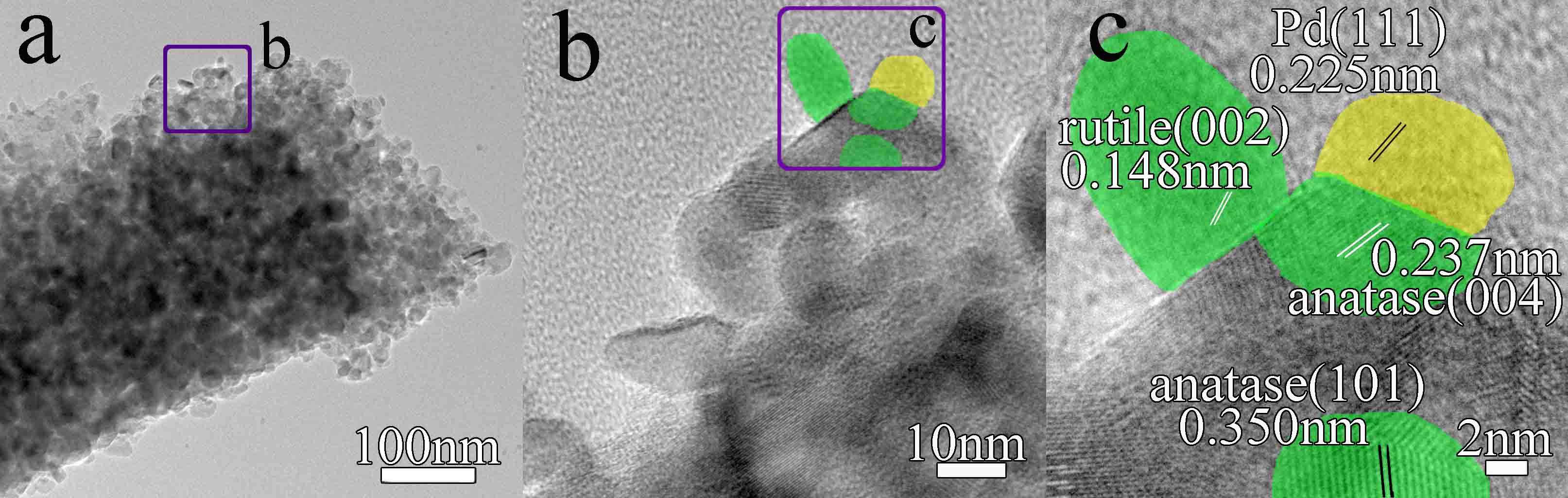


**Figure S4.** (a) TEM image of pPTND3 nanofibers after calcination, (b, c) HRTEM images with indicated d-spacings of pPTND3.


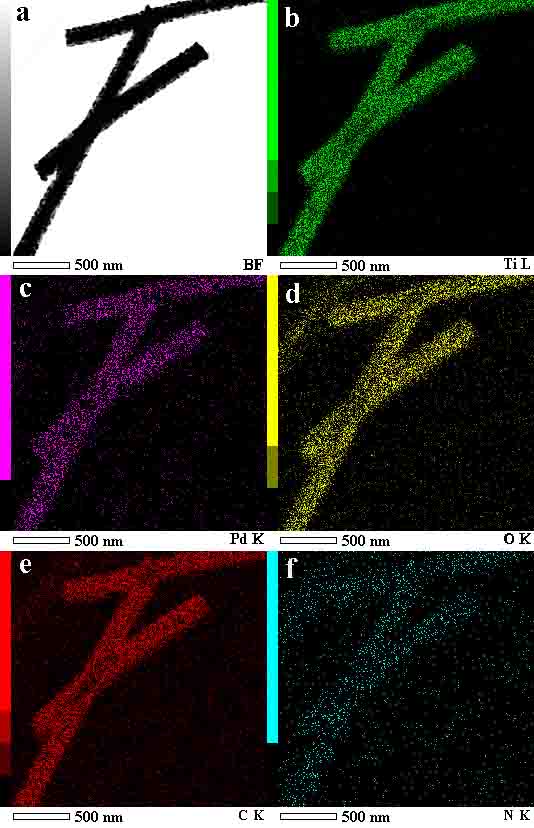


**Figure S5.** STEM image/EDS mapping of the PTND3. (a) bright field image, (b-f) corresponding to Ti, Pd, O, C and N elemental mapping, respectively.


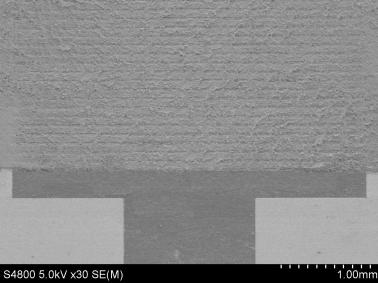


**Figure S6.** SEM image of the sensor coated with the composite thin film.

Thin film sensor：

1 mg sample was dispersed in 1 mL ethanol to obtain a suspension, and 50 uL suspension was dropped using a pipette onto the Au electrode, then dried at 60 ^o^C for 5 h to obtain thin film gas sensor.


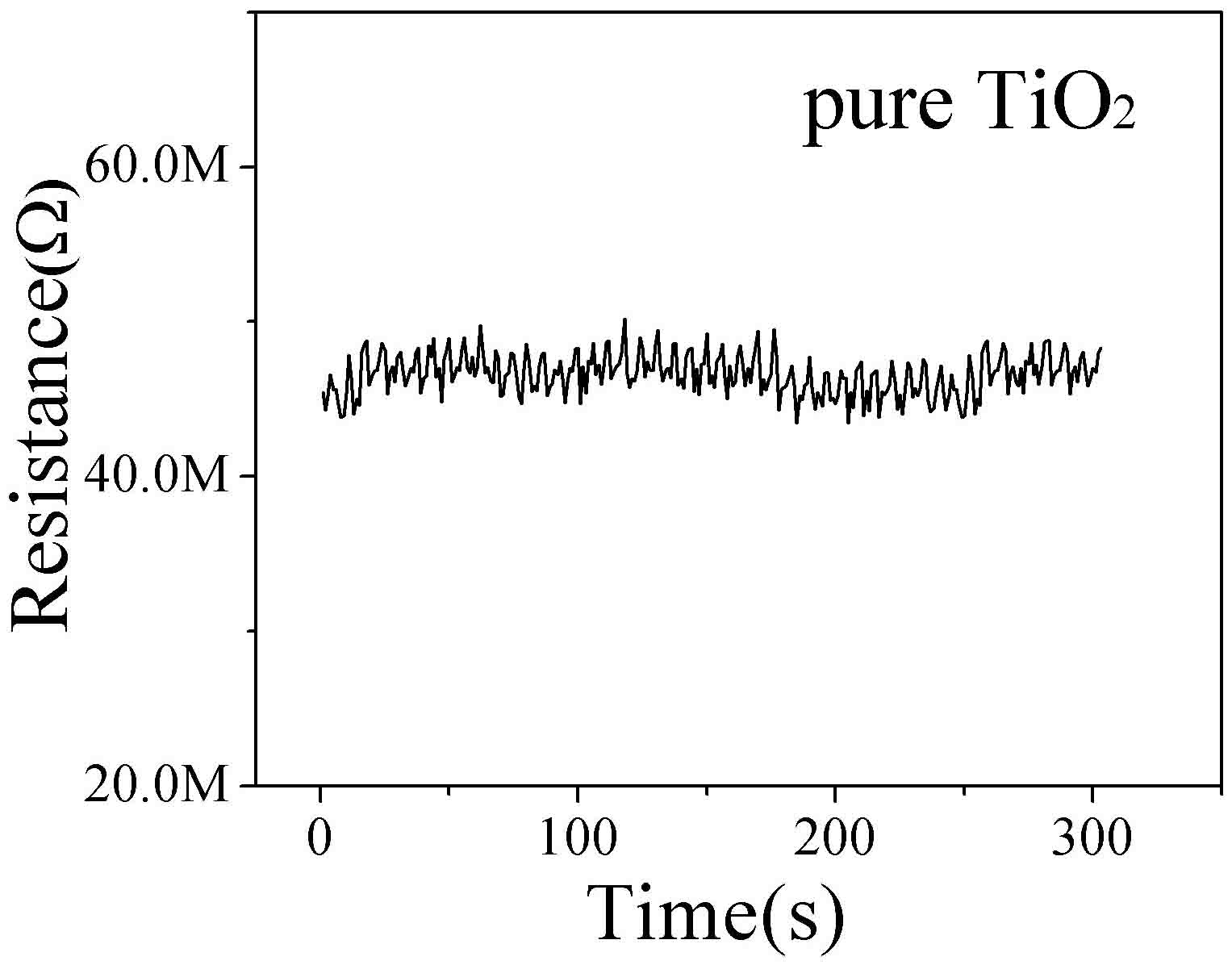


**Figure S7.** Dynamic response of the pure TiO_2_ sensor to 100 ppm NH_3_ concentrations at 26 ºC ( humidity 30%).


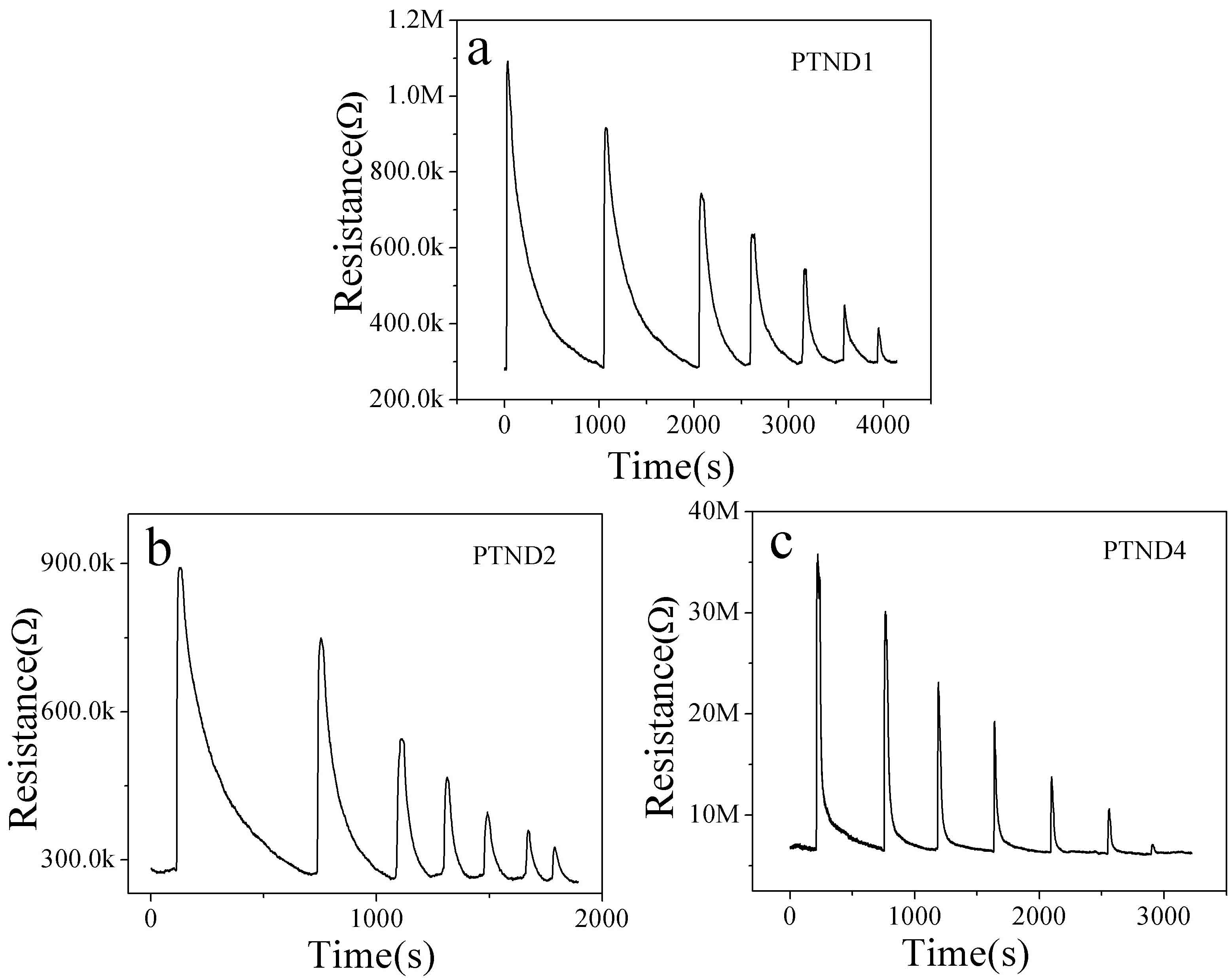


**Figure S8.** Dynamic response of the PTND1, PTND2 and PTND4 sensors to different NH_3_ (100, 50, 10, 5, 1, 0.5, 0.1 ppm) concentrations (at 26 ºC, humidity 30%).

**Table S3.** Response and response time of four samples (at 26 ºC, humidity 30%)

| Gas concentration  (ppm) | PTND1 | | PTND2 | | PTND3 | | PTND4 | |
| --- | --- | --- | --- | --- | --- | --- | --- | --- |
|  | Response | Response  time(s) | Response | Response  time(s) | Response | Response time(s) | Response | Response time(s) |
| 100 | 2.88 | 18 | 4.15 | 12 | **6.97** | **3** | 2.19 | 19 |
| 50 | 2.22 | 26 | 3.63 | 10 | **5.47** | **4** | 1.71 | 18 |
| 10 | 1.67 | 20 | 2.42 | 10 | **4.86** | **7** | 1.05 | 18 |
| 5 | 1.39 | 24 | 2.00 | 6 | **3.35** | **9** | 0.75 | 18 |
| 1 | 1.04 | 23 | 1.18 | 7 | **2.10** | **10** | 0.50 | 19 |
| 0.5 | 0.74 | 14 | 0.71 | 8 | **1.56** | **11** | 0.35 | 19 |
| 0.1 | 0.54 | 14 | 0.16 | 9 | **1.00** | **14** | 0.26 | 16 |

**Table S4.** Response of pPTND3 (at 26 ºC, humidity 30%)

| Gas concentration (ppm) | 100 | 50 | 10 | 5 | 1 | 0.5 |
| --- | --- | --- | --- | --- | --- | --- |
| Response | 1.43 | 0.92 | 0.66 | 0.51 | 0.24 | 0.08 |
| Response time (s) | 32 | 39 | 66 | 31 | 27 | 25 |


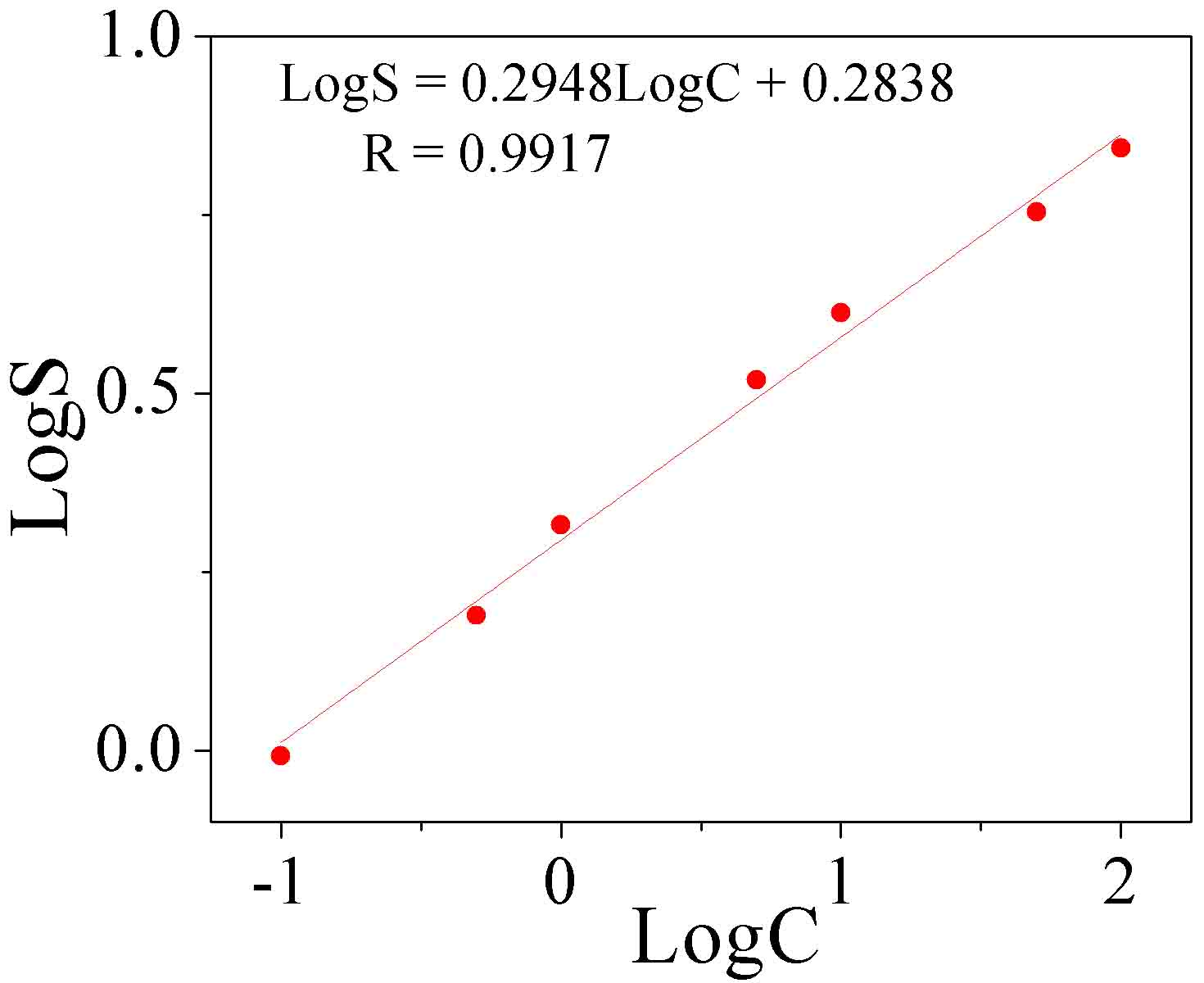


**Figure S9.** The linear range of NH_3_ response of PTND3 (at 26 ºC, humidity 30%).


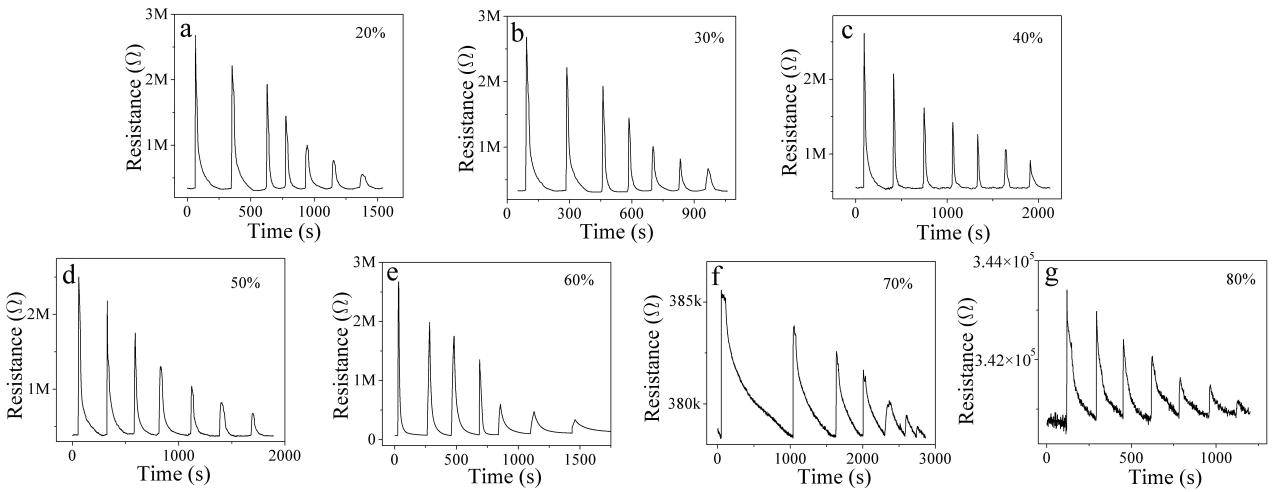


**Figure S10.** The results of the gas response of sensors on different humidity.

(a) humidity at 20%, (b) humidity at 30%, (c) humidity at 40%, (d) humidity at 50%, (e) humidity at 60%, (f) humidity at 70%, (g) humidity at 80%.


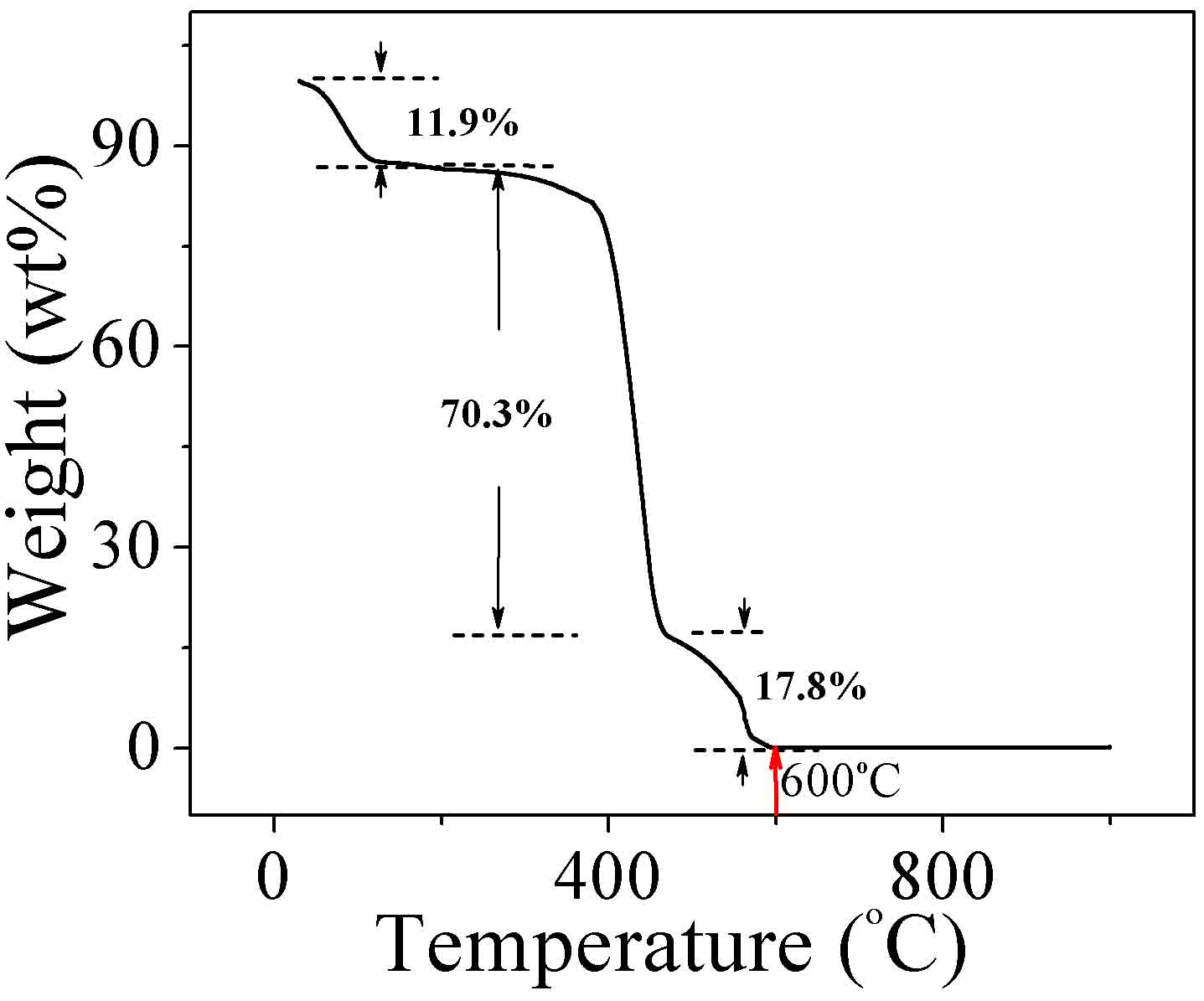


**Figure S11.** TG curve of PVP nanofibers (sample was heated at 10 ºC min^-1^ under air).


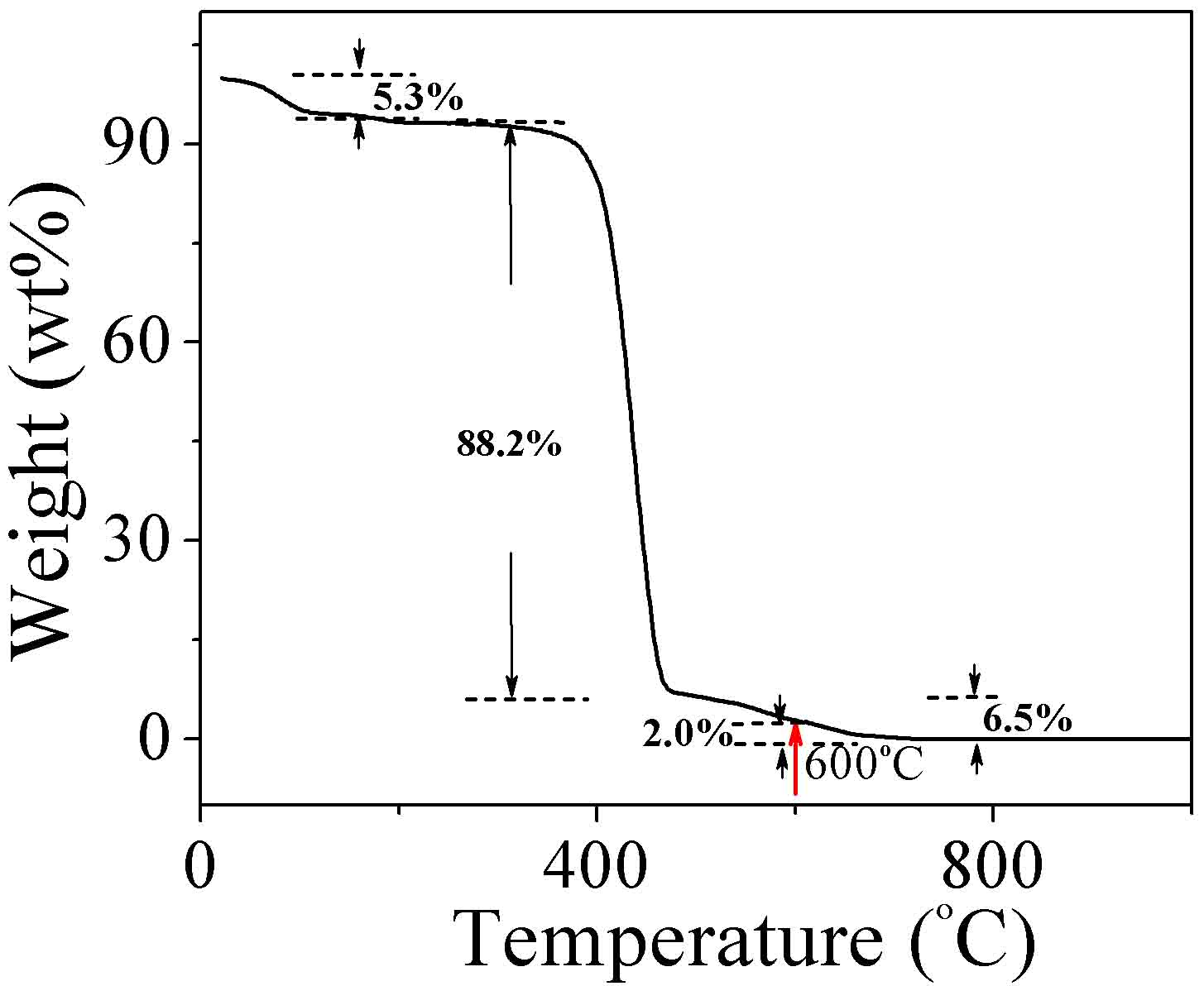


**Figure S12.** TG curve of PVP nanofibers (sample was heated at 10 ºC min^-1^ under mixture atmosphere of N_2_ and air).


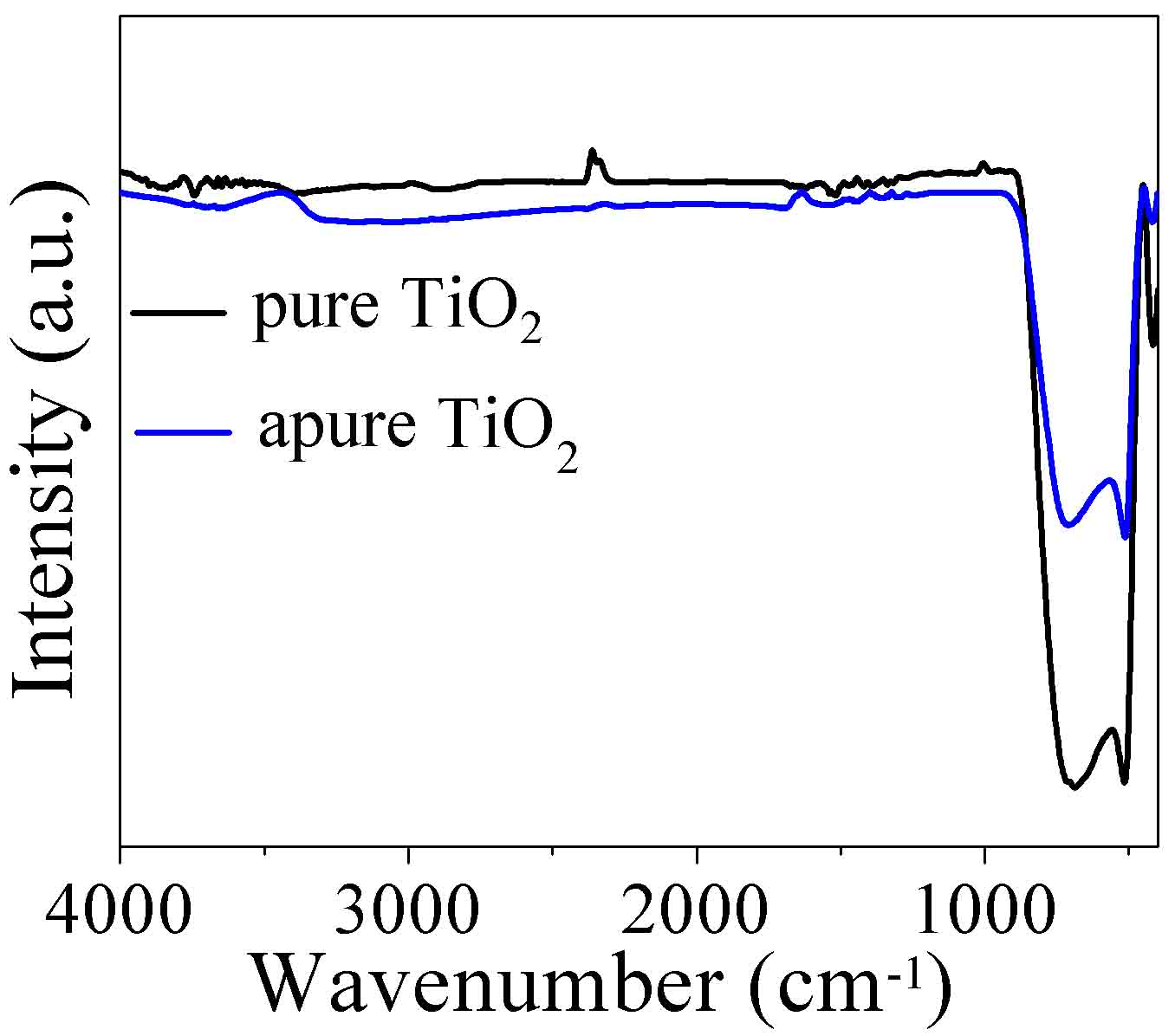


**Figure S13.** IR spectra curves of pure TiO_2_ and apure TiO_2_ (pure TiO_2_ adsorbed NH_3_).

Fig. S13 displayed the IR spectra curves of pure TiO_2_ and apure TiO_2_ (pure TiO_2_ adsorbed NH_3_) at 26 ºC. It can be observed, neither bands at 3340 and 1600 cm^-1^ nor bands at 3230 and 1470 cm^-1^ appear in the IR spectra of apure TiO_2_. There is only characteristic peak of Ti-O at 484 cm^-1^. So, it deduced there was no interaction of NH_3_ and pure TiO_2_. That means, neither Lewis acid sites nor Brønsted acid sites are existing in apure TiO_2_, since there are no Pd NPs or 16.2% residual of elements of C, N in apure TiO_2_.


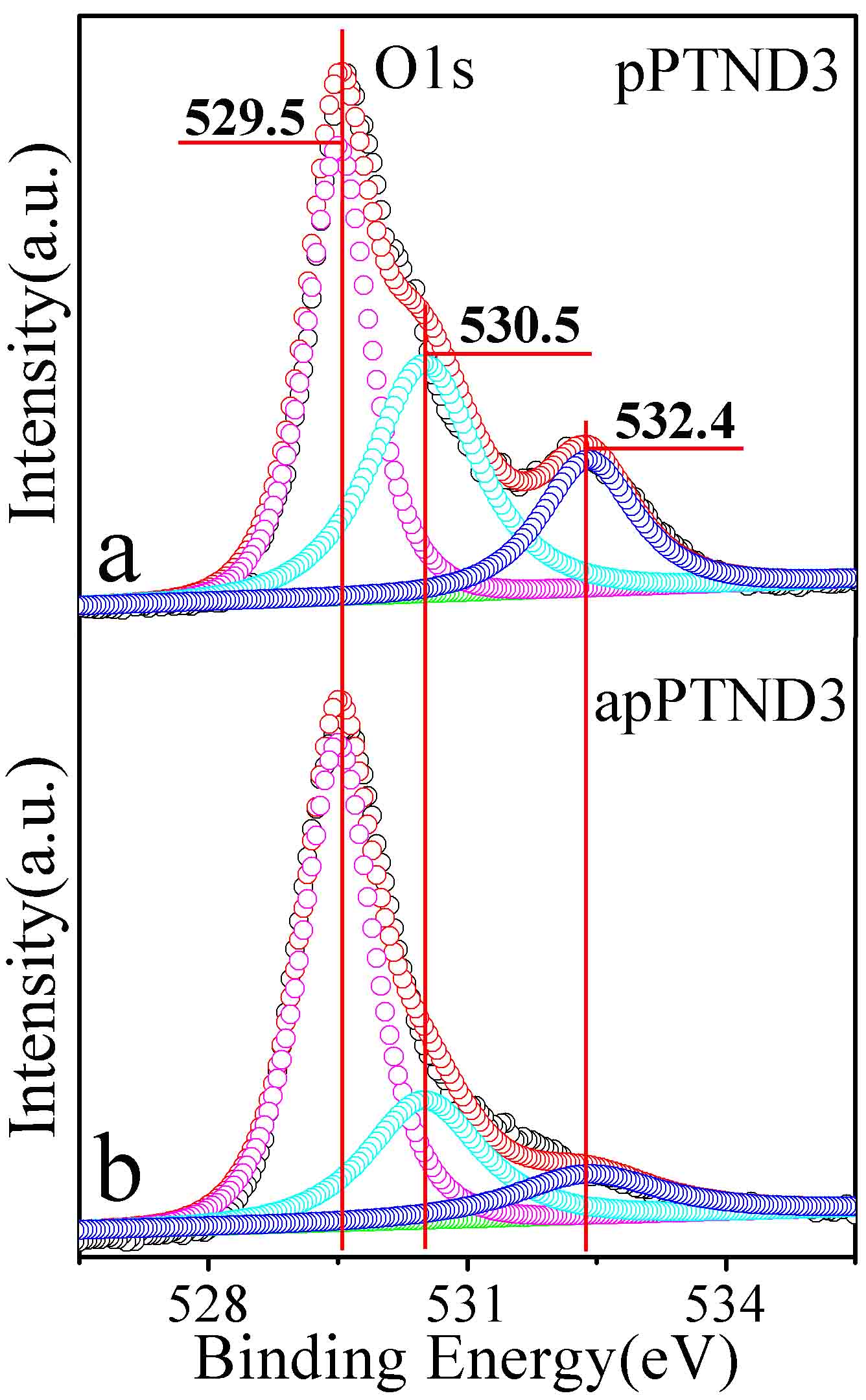


**Figure S14.** (a), (b) Curve-fitted XPS spectra of O 1s of pPTND3 and apPTND3.


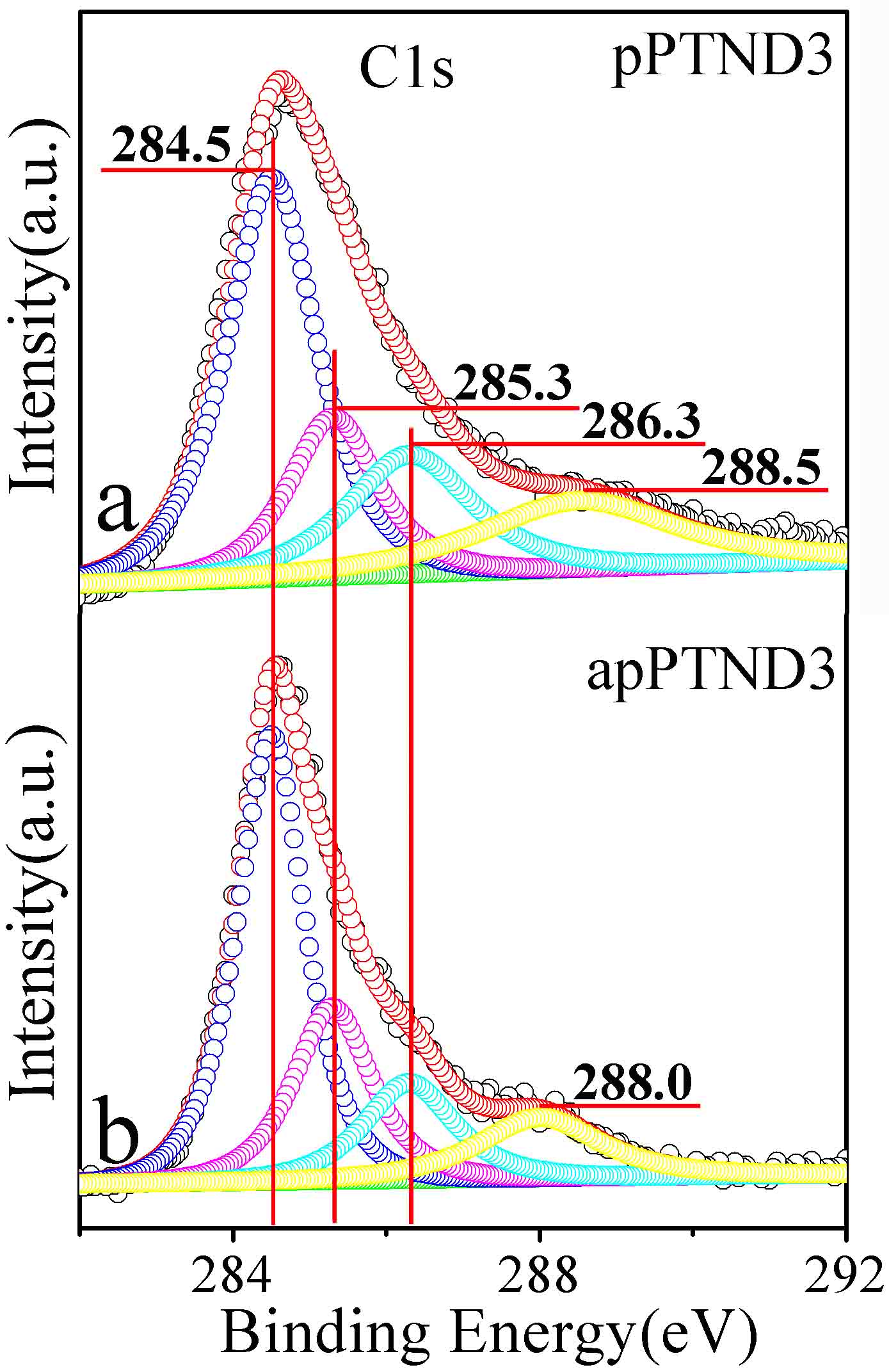


**Figure S15.** (a), (b) Curve-fitted XPS spectra of C 1s of pPTND3 and apPTND3.


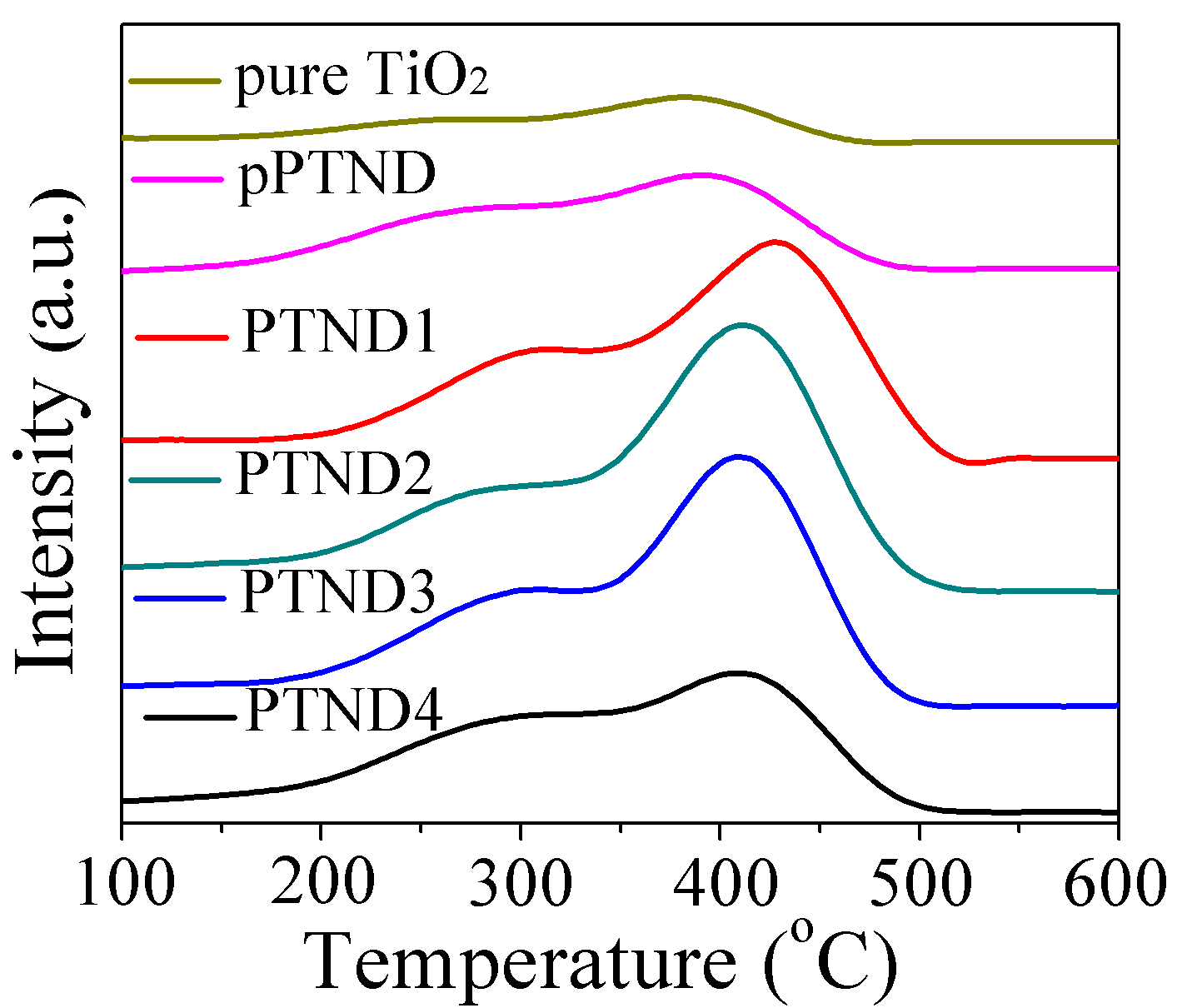


**Figure S16.** NH_3_-TPD profiles of pure TiO_2_, pPTND, PTND1, PTND2, PTND3 and PTND4.
